# Gender Dependent Evaluation of Autism like Behavior in Mice Exposed to Prenatal Zinc Deficiency

**DOI:** 10.3389/fnbeh.2016.00037

**Published:** 2016-03-03

**Authors:** Stefanie Grabrucker, Tobias M. Boeckers, Andreas M. Grabrucker

**Affiliations:** ^1^Institute for Anatomy and Cell Biology, Ulm UniversityUlm, Germany; ^2^WG Molecular Analysis of Synaptopathies, Neurology Department, Neurocenter of Ulm UniversityUlm, Germany

**Keywords:** Zn, Zn^2+^, ZnT, ASD, brain, plasticity, ZIP

## Abstract

Zinc deficiency has recently been linked to the etiology of autism spectrum disorders (ASD) as environmental risk factor. With an estimated 17% of the world population being at risk of zinc deficiency, especially zinc deficiency during pregnancy might be a common occurrence, also in industrialized nations. On molecular level, zinc deficiency has been shown to affect a signaling pathway at glutamatergic synapses that has previously been identified through genetic mutations in ASD patients, the Neurexin-Neuroligin-Shank pathway, via altering zinc binding Shank family members. In particular, prenatal zinc deficient but not acute zinc deficient animals have been reported to display autism like behavior in some behavioral tests. However, a full behavioral analysis of a possible autism like behavior has been lacking so far. Here, we performed an extensive behavioral phenotyping of mice born from mothers with mild zinc deficiency during all trimesters of pregnancy. Prenatal zinc deficient animals were investigated as adults and gender differences were assessed. Our results show that prenatal zinc deficient mice display increased anxiety, deficits in nest building and various social interaction paradigm, as well as mild alterations in ultrasonic vocalizations. A gender specific analysis revealed only few sex specific differences. Taken together, given that similar behavioral abnormalities as reported here are frequently observed in ASD mouse models, we conclude that prenatal zinc deficient animals even without specific genetic susceptibility for ASD, already show some features of ASD like behavior.

## Introduction

Although in industrialized nations micronutrient deficiencies occur rarely in pregnant women at levels of severity that may lead to obvious abnormalities in their infants, zinc may be an exception (Sandstead et al., 1977). Especially the fetal brain, because of the nature of its growth and development, seems particularly vulnerable to maternal zinc deficiency.

Apart from the many signaling and metabolic pathways in the central nervous system (CNS) that zinc is involved in, zinc has a particular role in neurogenesis, neuronal migration and differentiation, and synaptic plasticity (Frederickson et al., 2000; Levenson and Morris, 2011; Takeda et al., 2013). In addition, zinc is a signaling ion in nerve cells and potent modulator of neurotransmission. Thus, zinc is crucial for brain development and it is therefore not surprising that prenatal zinc deficiency results in impairments in cognitive performance and behavior later in life. Especially in animal studies, prenatal zinc deficiency was reported to result in altered emotionality, altered states of anxiety, and aggression, as well as altered social behavior and impaired memory and learning later in life (reviewed in Hagmeyer et al., 2015). However, despite the similarity of phenotypes described, no direct association to autism spectrum disorders (ASD) has been made, probably given that most of these studies have been conducted between 1970 and the early 1980s.

Intriguingly, already in 1976, it was noted that mood disturbances occurring in *Acrodermatitis enteropathica* (AE) patients (patients suffering from mostly hereditary zinc deficiency i.e., due to mutations in zinc uptake transporters such as ZIP4) that are particularly evident in infant and young patients, are often described as “schizoid” and that children with AE display some features similar to autistic children (Moynahan, 1976) such as avoidance of eye contact. Nowadays, many studies on human patients report zinc deficiency to occur frequently associated with neuropsychiatric disorders such as ASD, Attention deficit hyperactivity disorder (ADHD), Mood Disorders such as Depression, and Schizophrenia (Pfaender and Grabrucker, 2014). Furthermore, zinc deficiency has been associated with the etiology of ASD as environmental risk factor (Grabrucker, 2012; Vela et al., 2015).

In our previous studies (Grabrucker et al., 2014), we could show that prenatal zinc deficiency influences a signaling pathway at glutamatergic synapses that has been identified to be associated with ASD based on genetic mutations found in ASD patients (Bourgeron, 2009; Huguet et al., 2013). In particular, we found the zinc dependent regulated and ASD associated Shank2 and Shank3 proteins (Grabrucker, 2014; Leblond et al., 2014) were decreased at synaptic contacts in the CNS of prenatal zinc deficient pups after birth. Additionally, we have shown significant impairments in ultrasonic vocalization in adult male mice exposed to prenatal zinc deficiency as well as reduced maternal behavior in adult female mice exposed to prenatal zinc deficiency, along with increased aggression in a maternal resident intruder test (Grabrucker et al., 2014). However, although these results hint toward an ASD like phenotype in prenatal zinc deficient animals, no full behavioral assessment was performed. Thus, here, we report a detailed behavioral characterization of prenatal zinc deficient animals regarding a possible ASD like phenotype. To that end, we have chosen state of the art test paradigms to evaluate the three core symptoms associated with ASD: aberrant reciprocal social interactions, repetitive behavior, as well as impairments in communication. Moreover, we evaluated the presence of features resembling co-morbidities often observed in human patients such as increased anxiety and mental retardation. All analyses were performed using a gender specific evaluation given the male to female sex ratio of at least 2–3:1 for autism in human patients (CDC, 2014; Halladay et al., 2015). In addition, data indicate that the zinc content of the brain might also show gender differences (Lee et al., 2002) and male and female offspring from prenatal zinc deficient mice might be differentially affected. For example, zinc deficiency may alter maternal testosterone levels (Om and Chung, 1996) due to excessive conversion of testosterone into estrogen by an aromatase that is normally inhibited by zinc.

## Materials and methods

### Generation of prenatal zinc deficient mice

Ten-weeks-old C3H/HenRj mice were purchased from Janvier Labs and housed upon arrival in the animal facility in plastic cages under standard laboratory conditions and provided with food and water available *ad libitum*. The housing room was maintained at 22°C, with lights automatically turned on/off in a 12 h rhythm (lights on at 7 am). After 1 week of acclimation, mice were divided into 2 groups, one group (8 females) was fed a zinc deficient diet (<5 ppm zinc, Ssniff diets, Germany) with distilled, demineralized drinking water, while the control group (8 females) was fed with standard laboratory food (35 ppm zinc) and tapped water. After 5 weeks, females of the control and zinc deficient group were mated. After birth, mice were transferred to and nursed by zinc adequate surrogate mothers from postnatal day (PD) 2 on. All animals used in the experiments were cross-fostered, including CTRLs. Behavioral testing of the offspring began at the age of 15 weeks. All experiments were conducted between 9 and 6 pm. At least 1 h before behavioral testing mice were habituated to the test room. All animal experiments were performed in compliance with the guidelines for the welfare of experimental animals issued by the Federal Government of Germany and the local ethics committee (Ulm University; ID Number: O.103 and 1257).

### Measurement of metal ion concentrations

The zinc-concentration of blood was measured by atomic absorption spectrometry (AAS) at the Department of Clinical Chemistry (ZE klinische Chemie) of the University Hospital Ulm. ICP-MS measurements were performed at the “Spurenanalytisches Laboratorium Dr. Baumann” (Maxhütte-Haidhof, Germany). For immunohistochemistry, cryosections from pups were thawed for 20 min and zinc staining was performed using 10 μM Zinpyr1 (Sigma Aldrich) for 1 h at RT. Sections of control and zinc deficient mice were imaged using an upright Axioscope microscope equipped with a Zeiss CCD camera (16 bits; 1280 × 1024 ppi) using Axiovision software (Zeiss). The signal intensity was quantified by measuring at least three optic fields of view using ImageJ 1.49o and controls were set to 100%.

### Behavioral analysis

Behavioral tests are presented in the order corresponding to the “results” part: (1) General health and neurological reflexes, & Grip Strength Test, (2) Open Field, (3) Elevated Plus Maze, (4) Nest building, (5) Sociability and Social Novelty, (6) Olfactory habituation/dishabituation, (7) Male-Male reciprocal social interactions & Female-Female reciprocal social interactions (8) Ultrasonic vocalizations during female-female interaction/male-male interaction, (9) Rotarod, (10) Y-maze test, and (11) Self grooming, (13) Marble Burying.

#### General health and neurological reflexes

Mice were first evaluated for general health using a modified SHIRPA Test protocol (Rogers et al., 1997). Furthermore, mice were tested for the visual placing reflex (forepaw extensions when lowered toward a visible surface) and for the ability to grasp a metal grip with forepaws and hindpaws. Muscle strength was measured using a Bioseb gripmeter (Bioseb, France) on forelimbs and all limbs. Each assay was repeated three times, and measurements were averaged.

#### Locomotion and activity

Activity and locomotion in a novel environment was assessed by a 30 min test session in an open field arena. The arena was illuminated by overhead white lighting (100 lux) and constructed of white Plexiglas. The tested mouse was allowed to freely explore for 30 min period a 50 × 50 cm open field arena (with 20 × 20 cm center zone). Total distance traveled, average velocity and the time spent in the center zone vs. time spent at the boarder zone, number of entries into center and boarder zone, as well as numbers of ambulations were quantified using Viewer 2 software (BIOSERVE GmbH, Bonn, Germany).

#### Elevated plus maze for anxiety-like behavior

Anxiety-related behavior was analyzed using the elevated-plus maze. The maze was made of two open and two enclosed arms (with 16 cm high walls) and a central platform (5 × 5 cm) positioned 60 cm above the floor. Every mouse was placed in the central platform, facing one of the closed arms and allowed to explore the setting for 10 min. The behavior of the subject mice was analyzed using Viewer 2 software (BIOSERVE GmbH, Bonn, Germany) in terms of time spent in, and entries in open arms and closed arms, average velocity and number of ambulations.

#### Nest pattern in the home cage

Mice were isolated for 1 week to allow habituation to the single housing. Three days before the test, a nestlet (5 × 5 cm, mean weight 2.6 g) was introduced in the home cage of the mice in order to allow habituation to the nestlet. The nesting material was not removed until the test day. On the test day, 2 h before the dark phase, the old nest material was removed and a new nestlet was placed in the home cage of the mouse. Nest building ability was assessed the following morning, according to a 5 point rating scale (Deacon, 2006).

#### Social approach and preference for social novelty

Adult social approach behavior and preference for social novelty was tested as previously described by Yang et al. (2011). The apparatus consisted of a rectangular three-chambered box with retractable doorways within the two dividing walls. A video camera (Conrad CCD camera S/W) was mounted over the rectangular chamber (36 × 20 × 20 cm) to allow recording of the sessions. Videos were digitized by Pinncacle Studio 500-PCI, version 10. The test was divided in three different phases (habituation, sociability and social novelty). In the habituation phase, the mouse was first allowed to freely explore the whole setting (both side compartments contained an empty wire cup) for a period of 10 min (phase 1). After the habituation period, an unfamiliar C3H/HenRj mouse of the same sex and age (stranger 1) was placed under one of the cups. The location of stranger 1 (left vs. right side) was counterbalanced between each subject. At the end of the 10 min sociability test, the subject mouse was restricted to the central compartment, while an unfamiliar mouse of the same sex (stranger 2) was placed under the cup of the other side. Each subject mouse was tested again for 10 min, in order to evaluate the social preference for a new stranger (phase 3). In all three phases, measures were taken of the amount of time spent in each chamber, and number of entries into each chamber using the tracking software EthoVision XT (Noldus, Wageningen, Netherlands). An entry was defined as the center point of the mouse being in one of the 3 chambers. The time spent sniffing each wire cage during the different test sessions was manually scored by a human observer with a stopwatch. Sniffing was defined as a clear nose contact with the wire cage. Between each subject, the arena was cleaned with 70% ethanol and wiped with dry paper and left 5 min in order to allow ethanol evaporation. Observations and recordings were made in a soundproof room. The animals serving as stranger mice, were group housed (2–3 mice per cage), sex and aged matched and obtained from Janvier Labs. Stranger mice had no previous physical contact with the test mouse and were habituated to the wire cage 3 days before the test session.

#### Olfactory habituation/dishabituation test

Given the essential role of rodents olfaction in communication and social behavior, we examined olfactory ability as well as interest in non-social vs. social odors in a standard olfactory habituation and dishabituation test (Yang and Crawley, 2009). The test was carried out in a sound proof chamber under dim red light (15 lux). Mice were first habituated 1 day before the test session for 10 min to the test cage, containing a sterile dry cotton swab in order to avoid object neophobia. Nonsocial (distilled water, almond, banana) and social odors (social 1, social 2) were presented on cotton swabs inserted in the test cage in a sequential series for a period of 2 min (intertrial interval: 1 min) in the following order: water, water, water; almond, almond, almond (1:100 dilution, almond extract, Ostmann, Germany), banana, banana, banana (1:100 dilution, Uncle Roys natural banana essence, Scotland), social 1, social 1, social 1 (from the bottom of a cage with an unfamiliar age and sex matched group of C3H/Henrj) social 2, social 2, social 2 (from the bottom of a cage with a different sex matched group of B6 mice). All non-social odors were presented after dipping the cotton swabs for 1–2 s in the prepared solutions, whereas social odors were obtained by wiping the cotton swabs in zigzag fashion on the cage bottom of group housed animals (4 mice per cage). Cages of the animals serving as “social odor mice” were not changed for 1 week in order to ensure odor potency. Sniffing was defined as contact of the nose with the applicator (≤2 cm).

#### Adult reciprocal social interaction and ultrasonic vocalizations in the resident intruder paradigm

To evaluate the effect of prenatal zinc deficiency on direct social interaction, adult male—male and female—female reciprocal interactions were analyzed in the home cage of the subject mice. Mice were housed individually for a period of 4 weeks to increase their social motivation (Chabout et al., 2012). For 9 days before testing, the cages of the mice were not cleaned and wood shavings were not removed. After a 10 min habituation period in the sound proof chamber, residents were exposed to an unfamiliar intruder mouse for a period of 6 min. Animals serving as intruder mouse were group housed (3–4 per cage) sex and age matched and had approximately the same weight as the resident mouse. Behavior was recorded in a sound proof chamber with dim red light (15 lux) and a video camera (Conrad CCD camera s/w) was mounted 20 cm above the home cage of the subject mouse and subsequently analyzed for a period of 4 min. A detailed analysis of social interactions parameters was carried out using ICY platform with the semiautomatic plugin MiceProfiler Software (de Chaumont et al., 2012). Social interaction sequences were scored after following parameters: (1) contact events (i, contact duration (threshold < 1 cm); ii, oral–oral; iii, oral genital; iv, side by side contact). (2) Relative position events. (3) Dynamic events. Aggressive behavior was analyzed manually by scoring the number of attacks (mice preforming sideway threats, circling behavior, bites on the dorsal region or flank of the intruder or pushing intruder with forepaws at anybody region).

During male—male and female—female social interactions, ultrasonic vocalizations were recorded simultaneously by a condenser microphone (CM16; Avisoft Bioacoustic, Berlin, Germany), sensitive to frequencies of 10–180 kHz, which was mounted 28 cm above the center of the home cage. Previous data shows that ultrasonic vocalizations are emitted predominantly by the resident (Chabout et al., 2012). Ultrasonic vocalizations were recorded with Avisoft recorder Software (UltraSoundGate 116 USB, version 3.2 Avisoft Bioacoustics, Berlin, Germany). The settings of the recorder software were set to a sampling frequency of 300 kHz and a 16 bit format. For further acoustical analysis, WAV file recordings were transferred to SASLab pro Software (version 4.5, Avisoft Bioacoustics) and a Fast Fourier transform (FFT) was conducted (512 FFT length, 100% Frame, Hamming window, time resolution: 75% overlap). The spectrogram was generated at 586 Hz frequency resolution and a time resolution of 0.4267 ms. Background noise was eliminated by cutting of frequencies lower than 15 kHz with a high pass filter. Sound parameters were analyzed during the first 2 min of reciprocal social interactions and included (1) latency to call, (2) total number of ultrasonic vocalizations (3) percentage of single calls (4) percentage of calls with frequency jumps (5) percentage of calls with exactly one frequency jump (6) percentage of calls with more than one frequency jump (complex calls) (7) percentage of calls with overtones and harmonics. Starting point of reciprocal social interaction and call analysis was defined by the intruder mouse having all four paws on the bottom of the test cage.

#### Accelerated rotarod performance

Mice were analyzed for motor learning on an accelerating rotarod (TSE Systems, Bad Homburg, Germany). Mice were carefully placed on a rotarod apparatus with 4 rpm for a habituation period of 30 s. Afterwards, the rotational speed was increased from 4 to 40 rpm within 5 min. Mice were given 4 trials with 45 min break between each trial per day. Mice were tested on 2 consecutive days for a total amount of 6 trials. The latency to fall off the rod was measured. Mice that fall of the rod in less than 10 s were given a second trial.

#### Y-maze

Spontaneous alternation behavior was assessed in a symmetrical Y Maze (3 arms, 40 × 9 cm with 16 cm high walls). Arms choices (all four paws entering one arm) were recorded, while mice were allowed to freely explore the Y-shaped labyrinth for a period of 5 min. Alternation was determined by recording the order of the visited arms (A, B, or C). An arm entry was defined as the mouse having all four paws into the arms. Overlapping triplets of 3 arm visits was counted as one complete spontaneous alternation. The SAB score was calculated after following formula: (number of spontaneous alternation)/(total number of arm visits – 2). In order to prevent odor traces between animals, the walls and bottom of the Y Maze were carefully cleaned with 70% ethanol and wiped out with clean with paper towels. Videos were recorded by and analyzed using Viewer 2 software (Bioserve GmbH, Bonn, Germany).

#### Self-grooming

Repetitive time spent self-grooming was measured in a soundproof room under dim red light (10 lux). Each mouse was placed into a transparent plastic cylinder (diameter 35 cm, height 40 cm), without bedding material. A video camera (Conrad CCD camera S/W) was mounted approximately 15 cm in front of the test arena to allow recordings from the lateral view. An inclined mirror was positioned behind the test cage so that the surface on the mouse body facing away from the camera could also be seen for grooming analysis. After a habituation period of 10 min, each mouse was scored with a stopwatch for accumulative time grooming each body region for a period of 10 min. The videos were digitized by Pinncacle Studio 500-PCI, version 10. Video recordings were played back using Ulead VideoSoftware version 7.0 (accuracy 40 ms).

#### Marble burying test

Mice were first habituated to the soundproof chamber under dim red light (10 lux) for a period of 15 min. Subsequently, the mouse was placed in a clean and empty cage (26.5 cm length × 20 cm width × 14 cm height), filled with 4 cm of fresh bedding and marbles with 1.2 cm diameter placed on the surface. After 30 min, the total number of marbles (>50% buried) was recorded.

#### Statistics

Data are depicted as mean with standard error of the mean (SEM). Two-Way ANOVA was used to identify treatment (PZD) or gender effects, or interaction between the two factors in the Shirpa test, open field, elevated plus maze, resident intruder test, ultrasonic vocalization, Y Maze, marble burying and self-grooming. If significant effects were found in the factor of treatment × gender interaction, *post hoc* comparisons were conducted using Bonferrroni's post-tests. For the automated three chamber social approach test, within group repeated measures ANOVA were used to compare time spent in the two sides of the chamber, with the factor of chamber side (novel mouse vs. empty wire cage). The time spent sniffing the novel mouse vs. the empty wire cage was similarly analyzed. Time in the center is depicted in the graphs for illustrative purpose only. Treatment and gender effects in the Rotarod, three chamber test, olfactory habituation test, and body weight (between age 4 and 12 weeks) were analyzed using three-way mixed ANOVA. Nest building was analyzed using Mann-Whitney-U test. Multiple group comparison was done by Kruskal Wallis analysis. Statistical analysis was preformed with SPSS version 20. Statistical tests were two tailed with a significance level of α ≤ 0.05. Statistically significant differences are indicated in the figures by ^*^p ≤ 0.05, ^**^p ≤ 0.01 and ^***^p ≤ 0.001. In same cases trends are indicated with “#.” As gender effects, only significant differences between PZD males and females are shown.

## Results

**Figure 1:**
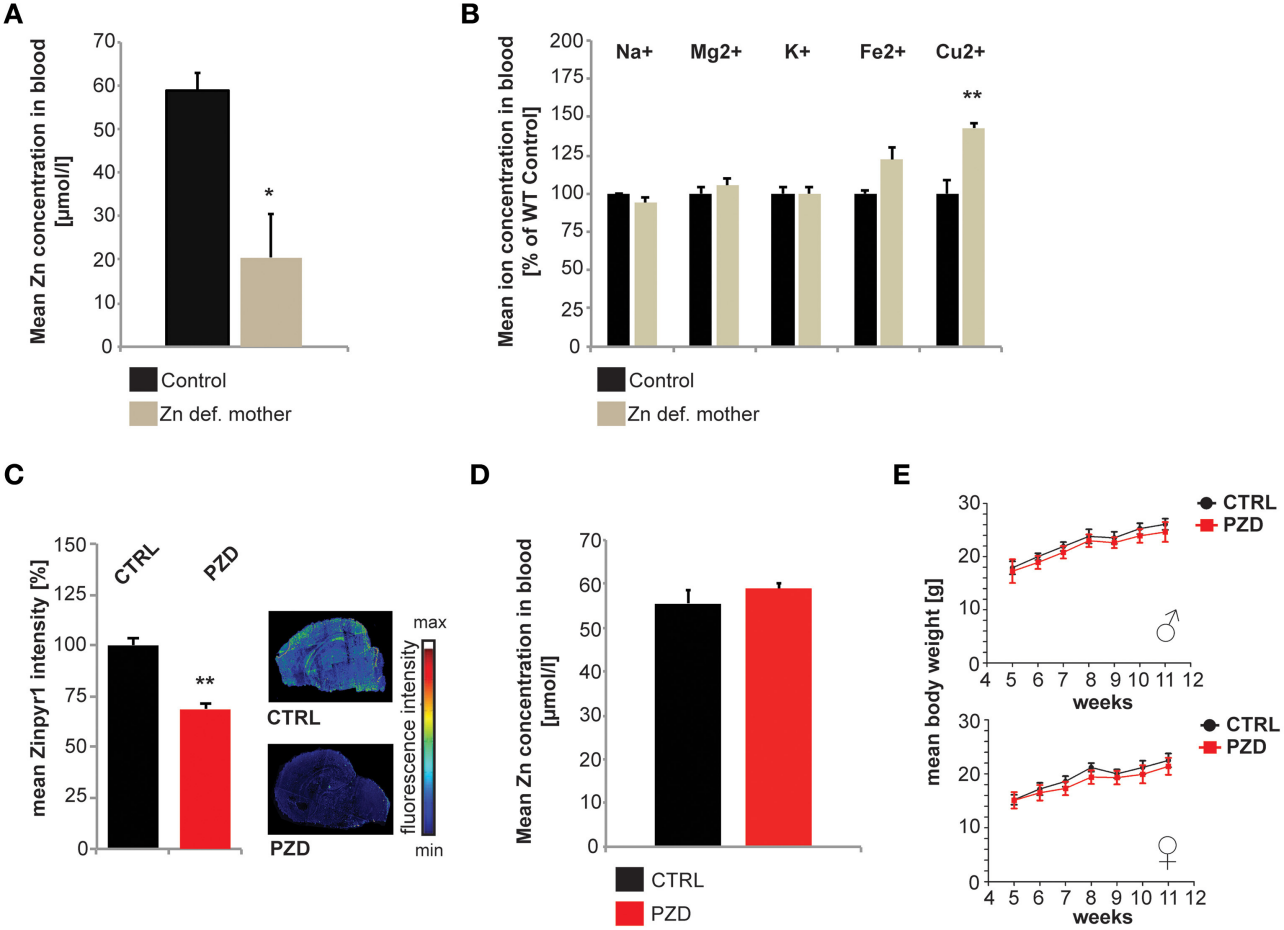
**Generation and general health of prenatal zinc deficient (PZD) mice. (A)** After 8 weeks of zinc deficient diet including 3 weeks of pregnancy, a significant reduction in blood zinc levels was found in zinc deficient mothers (*n* = 3 per group) using AAS. **(B)** Eight weeks of zinc deficient diet including 3 weeks of pregnancy did not significantly alter Na^+^, Mg^2+^, K^+^, and Fe^2+^ levels. However, the blood Cu^2+^ concentration was significantly (*p* = 0.009) increased (*n* = 3 per group; measured by ICP-MS). **(C)** Maternal zinc deficiency reduces the level of zinc in the brain of pups. Using the zinc specific fluorophore Zinpyr1, a significant reduction in signal intensity in brains from pups (post-natal Day PD2, *n* = 3) from zinc deficient mothers can be seen. Sections of control and PZD pups were imaged and quantified by measuring at least three optic fields of view and controls were set to 100%. **(D)** Blood zinc levels of adult PZD mice vs. Controls (*n* = 10 each). At the time-point of testing, no significant difference in blood zinc level was detected using AAS. **(E)** PZD mice displayed significant differences regarding their body weight compared to controls. *N* = 20 CTRL animals (10 males and 10 females) and *n* = 22 PZD mice (11 males and 11 females) were analyzed. ^*^p ≤ 0.05; ^**^p ≤ 0.01.

Since it was shown in the past that severe maternal zinc deficiency imposed by diets containing less than 1 mg Zn/kg/day has teratogenic effects and resulted in gross anatomical abnormalities in the offspring (Rogers et al., 1985), here we induced a mild maternal zinc deficiency throughout pregnancy. This lead to a significant reduction in blood zinc levels in mothers fed the zinc deficient diet during pregnancy (Figure 1A) and a significant increase in blood copper levels (Figure 1B). No alterations were observed regarding Na^+^, K^+^, Mg^2+^, and Fe^2+^ (Figure 1B). In the offspring of pregnant females on zinc deficient diet, we detected significantly reduced brain zinc levels (Figure 1C). The offspring of zinc deficient females did not show gross anatomical malformations. Although in humans, 20% of perinatal mortality worldwide has been attributed to zinc deficiency (Nriagu, 2007), the birth rate of zinc deficient (ZD) mice was comparable to controls (ZD: 5.6 ± 0.6; Ctrl: 5.1 ± 0.65; *n* = 10 pregnant animals per group). It was shown before that prenatal zinc deficient (PZD) pups quickly recover from prenatal zinc deficiency within days (Grabrucker et al., 2014). Thus, not surprisingly, at the age of behavioral testing, PZD animals displayed normal zinc levels (Figure 1D). Control (CTRL) and PZD mice were not significantly different regarding their general health, motor coordination, muscle tone, and neurological reflexes. However, female PZD mice show significantly increased freezing behavior after transfer (transfer arousal) (Table 1) hinting toward a possible increased anxiety (for statistical analysis see Supplementary Information 1). Additionally, PZD mice showed a significant reduction in body weight measured between the age of 4–12 weeks (Figure 1E) (three-way rmANOVA with age as a repeated measure. Main effect of the treatment: *F*_(1, 38)_ = 10.511, *p* < 0.003, main effect of the gender: *F*_(1, 38)_ = 96.826, *p* < 0.001, main effect of age: *F*_(6, 228)_ = 604.866, *p* < 0.001, treatment × age interaction: *F*_(6, 228)_ = 2.456, *p* < 0.044, gender × age interaction: *F*_(6, 228)_ = 5.279, *p* < 0.001).

**Table 1:** **General health of prenatal zinc deficient (PZD) mice**.

	**Male**		**Female**		
	**Control**	**PZD**	**Control**	**PZD**	
Number of animals (*n*)	10	11	10	11	
Weight [g]	26.7 (±0.21)	24.9 (±0.51)	22.4 (±0.58)	20.8 (±0.35)	
Body position	0.45 (±0.16)	0.55 (±0.16)	0.65 (±0.15)	0.36 (±0.15)	0 = inactive; 1 = active; 2 = excessive active
Tremor	0	0	0	0	0 = absent; 1 = present
Palpebral closure	0	0	0	0	0 = eyes open; 1 = eyes closed
Coat appearance	0	0	0	0	0 = tidy and well groomed; 1 = irregularities such as piloerection
Whiskers	0	0	0	0	0 = present; 1 = absent
Defecation	0.1 (±0.1)	0.27 (±0.14)	0.4 (±0.16)	0.27 (±0.14)	0 = present; 1 = absent
Transfer arousal	1.2 (±0.25)	0.72 (±0.24)	1.6 (±0.16)	0.64 (±0.2)	0 = extended freeze (over 5 s); 1 = brief freeze followed by movement; 2 = immediate movement
Locomotor activity	18.1 (±1.37)	16.2 (±2.21)	17.2 (±1.06)	16.5 (±1.73)	total number of squares crossing the animal enters with all four feet in 30 s
Gait	0.05 (±0.05)	0	0	0	0 = fluid movement (approx. 3 mm pelvic elevation); 1 = lack of fluidity in movement (more than 3 mm pelvic elevation)
Tail elevation	0.05 (±0.05)	0	0	0.27 (±0.14)	0 = dragging; 1 = horizontal extension; 2 = elevated/straub tail
Startle response	1 (±0.15)	1.18 (±0.12)	1	1.18 (±0.12)	0 = none; 1 = prayer reflex; 2 = reaction in addition to the prayer reflex
Touch escape	1 (±0.21)	1.18 (±0.18)	0.6 (±0.22)	0.81 (±0.23)	0 = no response; 1 = response to touch; 2 = flees prior to touch
Grip strength forepaw	4.04 (±0.26)	4.03 (±0.14)	4.56 (±0.23)	4.21 (±0.18)	grip strength per body weight
Grip strength all paw	7.85 (±0.31)	7.88 (±0.48)	7.65 (±0.30)	7.42 (±0.39)	grip strength per body weight
Positional passivity	0.4 (±0.07)	0	0	0	0 = struggles when held by the tail; 1 = struggles when held by the neck; 2 = struggles when laid supine; 3 = no struggle
Skin color	0.5	0.5	0.55 (±0.05)	0.5	0 = blanched; 1 = pink; 2 = bright, deep red flush
Trunk curl	0	0	0	0	0 = absent; 1 = present
Limb grasping	0	0	0	0	0 = absent; 1 = present
Pinna reflex	0	0	0	0	0 = present; 1 = absent
Corneal reflex	0	0	0	0	0 = present; 1 = absent
Contact righting reflex	0	0	0	0	0 = present; 1 = absent
Evidence of biting	0.1 (±0.1)	0	0	0	0 = none; 1 = biting in response to handling; 2 = excessive activity
Vocalization	0.3 (±0.15)	0.27 (±0.14)	0.4 (±0.16)	0.27 (±0.14)	0 = none; 1 = vocal

*Shirpa test. Control and PZD mice do not show significant differences in general health, motor coordination, locomotor activity or reflexes. Muscle strength was measured using a Bioseb gripmeter on forelimbs and all limbs. No significant differences were detected. Female PZD mice show significantly increased freezing behavior after transfer (transfer arousal). Data are expressed as means ± SEM*.

To investigate a possible relation of prenatal zinc deficiency and the development of neuropsychiatric symptoms later in life, we performed several behavioral tests. Mainly, we aimed at detecting a possible ASD-like phenotype in PZD mice. ASD is characterized by three core features in humans: impaired social interaction, impaired communication, and stereotyped behaviors and restricted interests. Furthermore, co-morbidities such as mental retardation, hyperactivity and increased anxiety occur frequently in ASD. Although the behavior of mice differs drastically from those of humans, features analogous to those seen in human ASD cases can be assessed. In the past, several paradigms demonstrated their validity in ASD mouse models with targeted deletion of ASD candidate genes (Ey et al., 2011; Chen et al., 2015). However, similar to the spectrum of phenotypes observed in humans, not all symptoms are present in these mice. Thus, a combination of assays is necessary to draw conclusions. We therefore tested PZD mice for core symptoms and co-morbidities of ASD using several test paradigms proposed by Crawley (Crawley, 2004).

### PZD mice show increased anxiety

**Figure 2:**
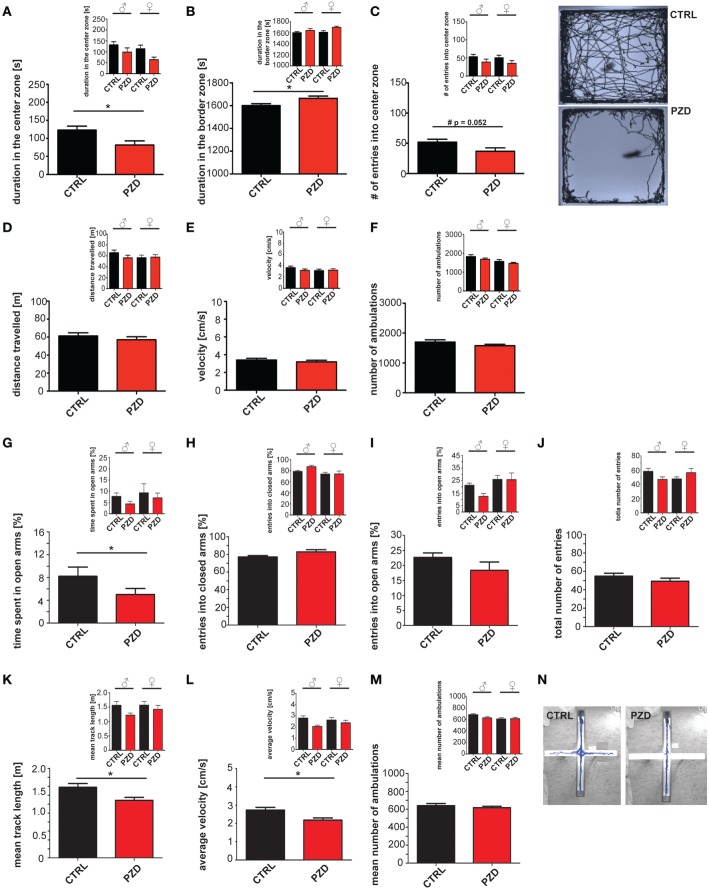
**PZD mice show increased anxiety**. Prenatal zinc deficiency affects anxiety related behaviors in the open field test **(A–F)** and elevated plus maze (EPM) **(G–N)**. **(A)** PZD mice spent significantly less time in the center of the open field arena and displayed increased time in the boarder zone **(B)**. Gender specific analysis (small inserts) shows a clear trend toward this behavior for both male and female PZD mice without significant gender specific effect. **(C)** PZD mice show a reduced number of entries in the center zone seen as clear trend (#). However, PZD mice are not impaired in the locomotor parameters of the open field test. **(D–F)** PZD mice walked the same distance, traveled with a speed similar to control mice, and showed no differences in the number of ambulations **(F)** compared to controls. **(A–F)** *N* = 20 CTRL animals (10 males and 10 females) and *n* = 22 PZD mice (11 males and 11 females) were analyzed. No gender specific effects were observed. **(G)** In the EPM, PZD mice show a significant difference to control mice regarding the time spent in the open arms of the maze. **(H)** The entries into the closed arms, and the entries into the open arms were not significantly different in PZD mice compared to controls. **(J)** The total number of entries was not significantly different in PZD mice. Regarding anxiety like behavior, no gender specific effect was observed. **(K–M)** In the EPM, PZD mice displayed impairments in the locomotor parameters. **(K)** PZD mice walked less a distance, and **(L)** traveled with less speed compared to control mice. **(M)** No significant differences were detected in the mean number of ambulations. **(N)** Exemplary images showing the tracked path of a CTRL and PZD mouse in the EPM. **(G–N)** *N* = 32 CTRL animals and *n* = 36 PZD mice were analyzed. ^*^p ≤ 0.05.

In a first set of experiments, we tested whether mice display anxiety related behaviors in the open field test (Figures 2A–F) and elevated plus maze (EPM) (Figures 2G–N). The open field test is a widely used procedure for examining locomotor activity and anxiety of mice (Choleris et al., 2001). Anxiety is an associated symptom of patients suffering on ASD (White et al., 2015) and autism mouse models display alterations in locomotor activity (Schmeisser et al., 2012). Our results show that, PZD mice spend significantly less time in the center of the open field arena (Figure 2A; two-way ANOVA, main effect of the treatment: *F*_(1, 38)_ = 7.049, *p* < 0.012) and remain mostly in the boarder zone (Figure 2B, two-way ANOVA, main effect of the treatment: *F*_(1, 38)_ = 5.495, *p* < 0.024). The locomotor parameters (distance walked (Figure 2D), velocity (Figure 2E), number of ambulations (Figure 2F)) were not impaired in PZD mice in the open field test. We did not observe any gender specific effects in the open field test.

To validate anxiety related behavior observed in the open field test we also measured anxiety like behavior in the more threatening arena of the EPM, which is based on the natural aversion of mice for open and elevated areas that competes with their natural spontaneous exploratory behavior in novel environments (Komada et al., 2008). Increased anxiety was also observed in this task, since PZD mice spend less time in the open arms of the maze (Figure 2G; two-way ANOVA, main effect of the treatment: *F*_(1, 64)_ = 5.335, *p* = 0.027). No significant difference was observed in the number of entries in the closed and open arms. The total number of entries was not significantly different. Furthermore, prenatal zinc deficiency causes reduced locomotor activity in the arena of the EPM. PZD mice traveled less distance (Figure 2K, two-way ANOVA, main effect of the treatment: *F*_(1, 64)_ = 4.989, *p* < 0.029) and displayed reduced velocity compared to control mice (Figure 2L, two-way ANOVA, main effect of the treatment: *F*_(1, 64)_ = 5.96, *p* < 0.017). These data suggest that increased anxiety is the main phenotype observed in this mouse model. No significant difference was detected in the number of ambulation in the open field arena.

### PZD animals show impaired nest building

**Figure 3:**
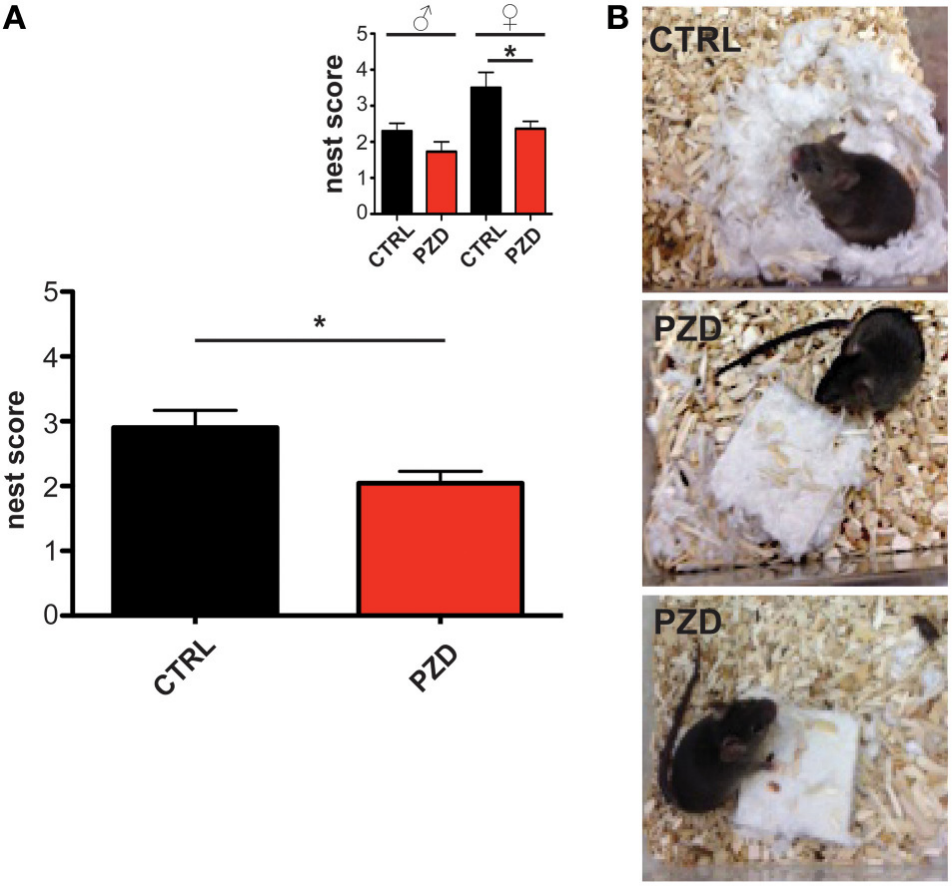
**PZD animals show impaired nest building. (A)** The nest quality score was assessed 24 h after introduction of a nesting material in the home cage according to a 5 point rating scale. PZD mice show deficits in nest building behavior. *N* = 20 CTRL animals (10 males and 10 females) and *n* = 22 PZD mice (11 males and 11 females) were analyzed. **(B)** Exemplary images showing the built nest after 24 h after introduction of nesting material from CTRL (upper image) and PZD (two lower images) mice. ^*^p ≤ 0.05.

For small rodents, nest building is important for heat conservation as well as reproduction and shelter. Additionally, the capacity to build a nest is associated with social behavior (Moretti et al., 2005; Deacon, 2006) and shown to be affected in several animal models for ASD, such as mice with deletions in Neurexin1α, Neuroligin1, PTEN, GABRB3, TSC1, and SHANK2 (Silverman et al., 2010; Jiang and Ehlers, 2013). Thus, we assessed the nest quality score 24 h after a nesting material was introduced in the home cage of mice (Figures 3A,B). PZD mice show significant deficits in nest building behavior compared to control mice (Figures 3A,B; *U* = 140, *p* < 0.023). Kruskal Wallis ANOVA revealed a significant difference among group (chi-square: 11.323, *df* = 3, *p* < 0.01). *Post hoc* comparison detected a significant difference between control female and PZD female mice (*p* = 0.049).

### PZD mice show altered social approach behavior and altered reciprocal social interactions

**Figure 4:**
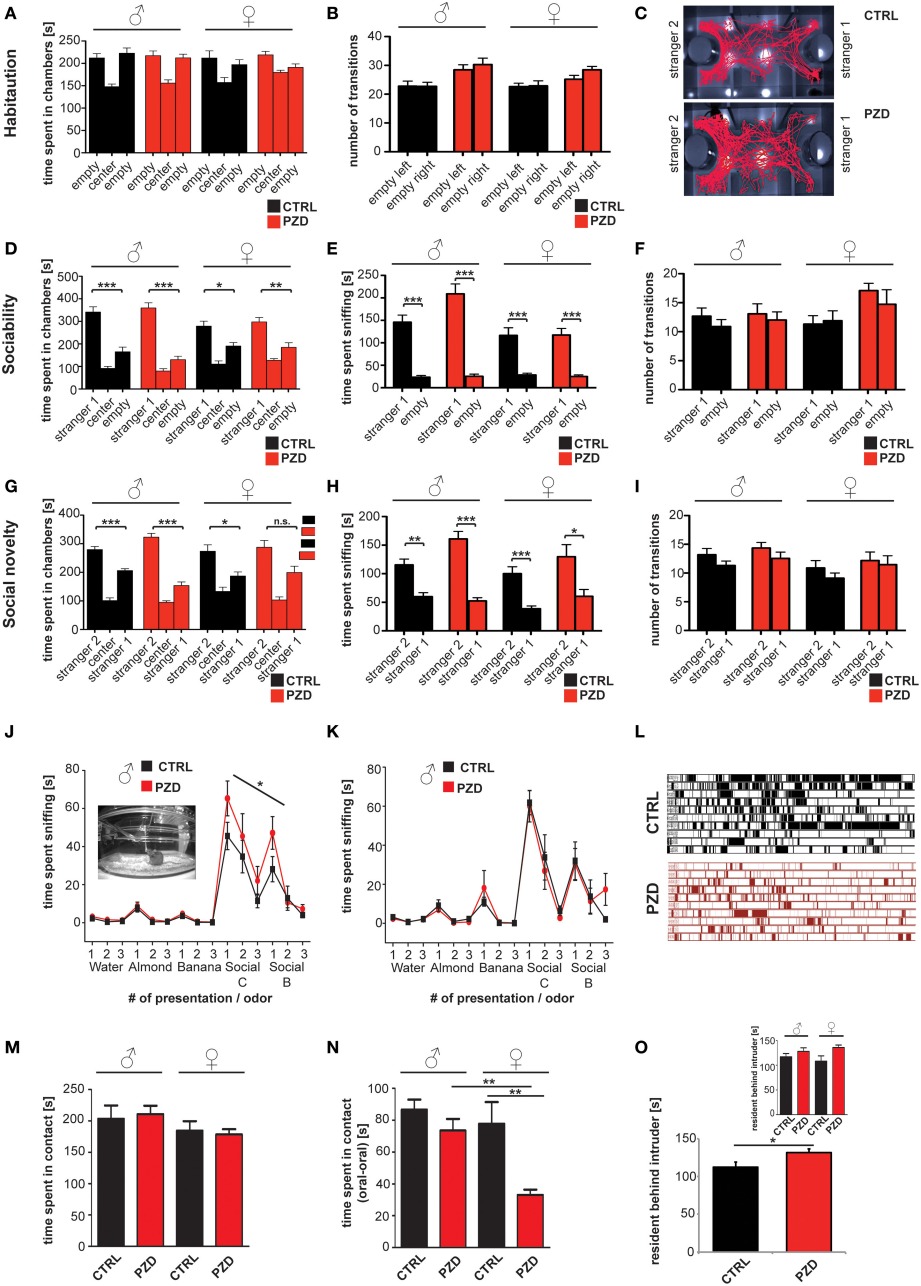
**PZD animals show altered behavior during the three chamber test, olfactory habituation test, and reciprocal social interactions. (A,B)** No innate side preference was detected in CTRL and PZD mice in the habituation phase given that no differences were detected in the time spent in a chamber the number of transitions **(B)**. **(C)** Exemplary tracking path of a CTRL and PZD mouse in the test for social novelty. **(D–F)** Normal sociability was found in PZD mice. Both, male and female PZD mice spent significantly more time in the chamber containing a stranger mouse vs. the empty wire cage **(D)** and sniffing the wire cage containing a stranger mouse **(E)**. **(F)** No significant difference in the number of transition was detected in this phase of the test. **(A–F)** No gender specific effects were detected. **(G–I)** In the second part of the test however, PZD male show a significant preference for stranger 2 in terms of time spent in the chamber with stranger 2 **(G)**, and the time spent sniffing at stranger 2 **(H)**. In contrast, female PZD mice do not display a significant preference for stranger 2 in terms of time spent in the chamber with stranger 2 **(G)**. **(H)** The time spent sniffing at stranger 2 was not altered in PZD female mice. **(I)** No significant difference in the number of transition was detected in this phase of the test. **(A–I)** *N* = 20 CTRL animals (10 males and 10 females) and *n* = 22 PZD mice (11 males and 11 females) were analyzed. **(J,K)** PZD mice were exposed to distilled water and four different odors consecutively presented three times for 2 min. The sniffing behavior of the animals in response to non-social odors: almond and banana extract, and social odors: animals of the same sex from the strains C3H/HeNRj (Social C1) and C57BL/6jRi6 (Social B1) was recorded and analyzed. The mean of the cumulative time the animals spent sniffing at the presented odor is presented. Abnormal sniffing times compared to controls can be seen regarding social odors in PZD males **(J)**. **(J,K)** *N* = 10 males and 10 females (CTRL) and *n* = 11 males and 11 females (PZD) were analyzed. **(L–O)** Reciprocal social interactions of male—male and female—female pairings were evaluated for 4 min. **(L)** Example chronogram representing all the labeled events of the data shown in **(B)** comparing female control and PZD mice over the 4 min of experimentation. Rows represent individual mice. Single events of contact are shown as lines. **(M)** The average time of contact between mice was quantified. No significant differences between CTRL and PZD were detected. **(N)** Oral—oral contact of mice was quantified and the average time spent in contact is shown. A significantly reduced contact time is seen specifically in PZD female mice compared to female controls and PZD male mice. **(O)** Increased aggression of PZD mice can be observed quantifying the average time of the resident chasing the intruder that is significantly increased for PZD mice without gender specific effect. **(L–O)** *N* = 20 CTRL animals (10 males and 10 females) and *n* = 22 PZD mice (11 males and 11 females) were analyzed. ^*^p ≤ 0.05; ^**^p ≤ 0.01; ^***^p ≤ 0.001.

To further assess social behavior, we performed a three-chamber assay. This test quantifies direct social approach behavior in mice and has strong face validity to approach behaviors observed in humans (Yang et al., 2011). The test is divided in three different phases (habituation, preference for sociability and preference for social novelty). Our results show that neither PZD nor control mice showed an innate side preference in the habituation phase. Thus, differences in the next phases cannot be explained be *a priori* preference of a specific chamber (Figures 4A,B).

In Phase 2, sociability of the subject mouse can be detected by measuring preference for spending time with a novel conspecific mouse in contrast to an empty chamber. Our results show, that after introducing a stranger mouse, both male and female PZD mice spent significantly more time in the chamber containing a stranger mouse vs. the empty wire cage and sniffing the wire cage containing the stranger mouse indicating the presence of sociability in PZD mice (Figures 4D,E, within group repeated measures ANOVA, factor chamber time (Figure 4D): [CTRL males: *F*_(1, 9)_ = 25.265, *p* < 0.001; PZD males: *F*_(1, 10)_ = 39.208, *p* < 0.001; CTRL females: *F*_(1, 9)_ = 6.518, *p* < 0.031; PZD females: *F*_(1, 10)_ = 10.935, *p* < 0.008], factor sniffing (Figure 4E): [CTRL males: *F*_(1, 9)_ = 41.884, *p* < 0.001; PZD males: *F*_(1, 10)_ = 45.577, *p* < 0.001; CTRL females: *F*_(1, 9)_ = 27.170, *p* < 0.001; PZD females: *F*_(1, 10)_ = 32.438, *p* < 0.001]). No significant effect was detected between PZD mice and CTRL mice in the time spent in the chambers during the sociability phase of the three chamber test (Figure 4D, three-way rmANOVA, main effect of chamber: *F*_(1, 38)_ = 74.725, *p* < 0.001, gender × chamber interaction: *F*_(1, 38)_ = 9.082, *p* = 0.005).

Altered approach behavior was observed in the third phase of the three chamber test, used to assess preference for social novelty and thereby social memory (Figure 4C). Our results show, that after the introduction of the second stranger, CTRL males: [*F*_(1, 9)_ = 24.425, *p* < 0.001], PZD males: [*F*_(1, 10)_ = 45.577, *p* < 0.001], and CTRL females: [*F*_(1, 9)_ = 6.279, *p* < 0.033] show a significant preference for the unfamiliar conspecific vs. the familiar one (Figure 4G, within group repeated measures ANOVA, factor chamber time). However, female PZD mice show no preference for the unfamiliar conspecific vs. the familiar one in the social novelty phase of the three chamber test, as measured by time spent in chamber [*F*_(1, 10)_ = 3.893, *p* < 0.077, Figure 4G, within group repeated measures ANOVA, factor chamber time].

However, a significant preference was detected for the factor of “sniffing” between all groups in the social novelty phase (Figure 4H, within group repeated measures ANOVA, CTRL males: *F*_(1, 9)_ = 20.136, *p* < 0.002; PZD males: *F*_(1, 10)_ = 43.260 *p* < 0.001; CTRL females: *F*_(1, 9)_ = 29.718, *p* < 0.001; PZD females: *F*_(1, 10)_ = 6.189, *p* < 0.035).

A significant treatment x gender interaction was detected in the time spent sniffing during the sociability phase (Figure 4E, three-way rmANOVA, main effect of the treatment: *F*_(1, 38)_ = 3.794, *p* < 0.059, main effect of the gender: *F*_(1, 38)_ = 13.171, *p* < 0.001; main effect of chamber: *F*_(1, 38)_ = 153.072, *p* < 0.001; treatment × gender interaction: *F*_(1, 38)_ = 4.418, *p* = 0.042; gender × chamber interaction: *F*_(1, 38)_ = 10.815, *p* = 0.002). *Post hoc* analysis revealed that PZD mice male mice displayed an increased time of sniffing the novel mouse (*p* < 0.05) vs. the empty wire cage in comparison to CTRL male mice. No significant differences were detected in the number of transitions during the sociability and social novelty phase (Figures 4F,I).

To ensure that PZD mice are able to detect social pheromones presented in the urin of conspecific mice that elicit social approach behavior as well as ultrasonic vocalizations, we preformed an olfactory habituation/dishabituation test (Yang and Crawley, 2009; Figures 4J,K). The task is used to assess olfaction, as well as interest in and recognition of social odors, two important components of rodent social interaction, and has been useful in clarifying mouse models of autism in the past. PZD mice showed normal olfactory habituation as indicated by a decline in time spent sniffing on repeated exposure to non-social or social odors (for detailed statistical results for habituation and dishabituation responses in each group, see Supplementary Information 1). PZD mice display dishabituation upon presentation of a new odor, as indicated by an increased time spent sniffing non-social or social odors. Interestingly, similar to the results in the three-chamber test, male PZD mice showed increased accumulative time spent sniffing compared to controls after presentation of the social odors (Figure 4J). Repeated measures ANOVA indicated that the peak height of sniff time across social odor trials (social C,B) was significantly higher in PZD males in comparison to control males [*F*_(1, 19)_ = 4.804, *p* < 0.042]. Interestingly, no significant difference was detected comparing the peak height of non-social odors (almond and banana) of PZD males to those of control males, indicating exaggerated response of PZD males toward social odors. No significant differences were detected in female PZD mice comparing peak height of social odors and non-social odors.

To further assess social behavior in PZD mice, particularly to investigate direct social contact events with conspecifics, we evaluated direct reciprocal male–male and female–female social interaction in the home cage of the subject mice (Figures 4L–O).

Although the average time the mice spent in contact was not significantly different between PZD and controls (Figure 4M), a significant difference during reciprocal social interactions could be detected in time spent in oral-oral contact in PZD mice in comparison to CTRL mice (Figure 4N; main effect of the treatment: *F*_(1, 38)_ = 15.103, *p* = 0.000; gender: *F*_(1, 38)_ = 8.352, *p* = 0.006; treatment × gender interaction: *F*_(1, 38)_ = 5.335, *p* = 0.027). *Post hoc* analysis revealed that specifically PZD females displayed a significant decrease in oral-oral contact (*p* < 0.004) in comparison to CTRL female controls. Additionally, there is a significant interaction between treatment × gender [*F*_(1, 38)_ = 5.335, *p* = 0.027]. Further, we could observe an increase in aggressive behavior that can be seen as increase in the average time of “resident behind intruder” events (Figure 4O, two-way ANOVA, main effect of the treatment: *F*_(1, 38)_ = 3.582, *p* < 0.038). These events were accompanied by aggressive postures and “chase-bites.”

### PZD mice show impaired communication, motor learning, and minor differences in repetitive behavior

**Figure 5:**
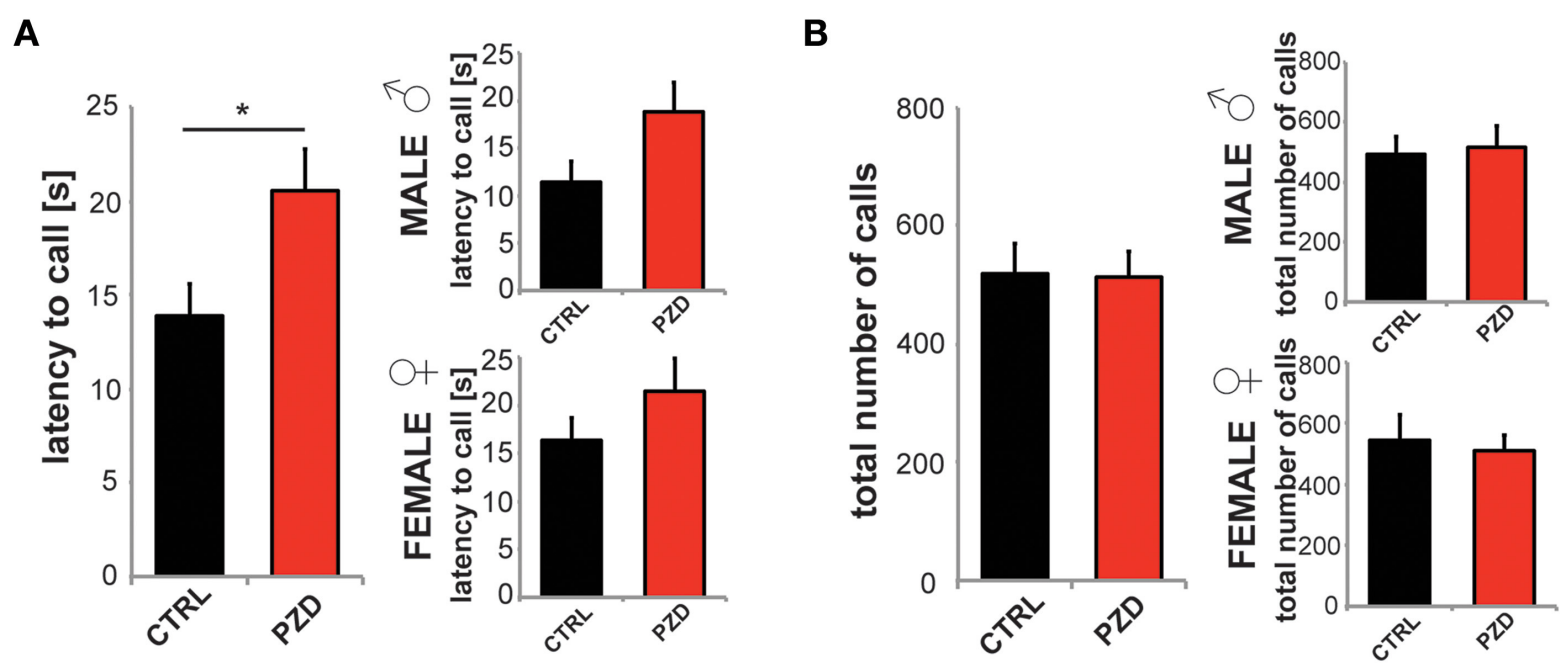
**PZD mice show altered vocalization**. Reciprocal social interactions of male—male and female—female pairings were evaluated for 4 min. **(A,B)** Ultrasonic vocalizations were recorded during the first 2 min of the test for reciprocal social interactions. **(A)** The latency to call was significantly increased in PZD animals. Gender specific analysis shows no gender specific effect. **(B)** The total number of calls was not reduced. *N* = 20 CTRL animals (10 males and 10 females) and *n* = 22 PZD mice (11 males and 11 females) were analyzed. ^*^p ≤ 0.05.

Further, we analyzed ultrasonic vocalizations of the mice during reciprocal social interactions. The latency to call was significantly increased in PZD animals (Figure 5A, two-way ANOVA, main effect of the treatment: *F*_(1, 38)_ = 5.215, *p* = 0.028). The total number of calls was not reduced (Figure 5B).

Next, we assessed motor learning and coordination in PZD mice using a rotarod test. Impaired motor learning was reported in several ASD mouse models (Moy et al., 2006) such as GABRB3, Ube3a (Angelman's syndrome) and NF1 (Neurofibromatosis type 1) mice.

**Figure 6:**
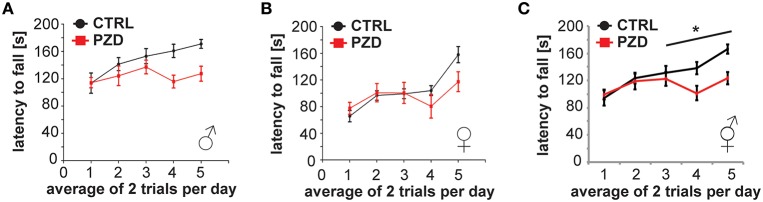
**PZD mice show impaired motor learning. (A–C)** Motor learning task using a Rotarod Mice were tested with two training trials per day. **(C)** The latency to fall was significantly different between CTRL and PZD mice. *N* = 22 CTRL animals and *n* = 24 PZD mice were analyzed. ^*^p ≤ 0.05.

PZD mice display impaired motor learning on the accelerated rotarod. PZD mice had consistently decreased latencies to fall from the rotarod apparatus in comparison to control mice (Figures 6A–C, three-way rmANOVA; main effect of the treatment: *F*_(1, 41)_ = 5.116, *p* < 0.029; main effect of gender: *F*_(1, 41)_ = 21.609, *p* < 0.001; main effect of trial: *F*_(4, 168)_ = 13.077, *p* < 0.001, treatment × trial interaction: *F*_(4, 168)_ = 3.750, *p* < 0.010).

**Figure 7:**
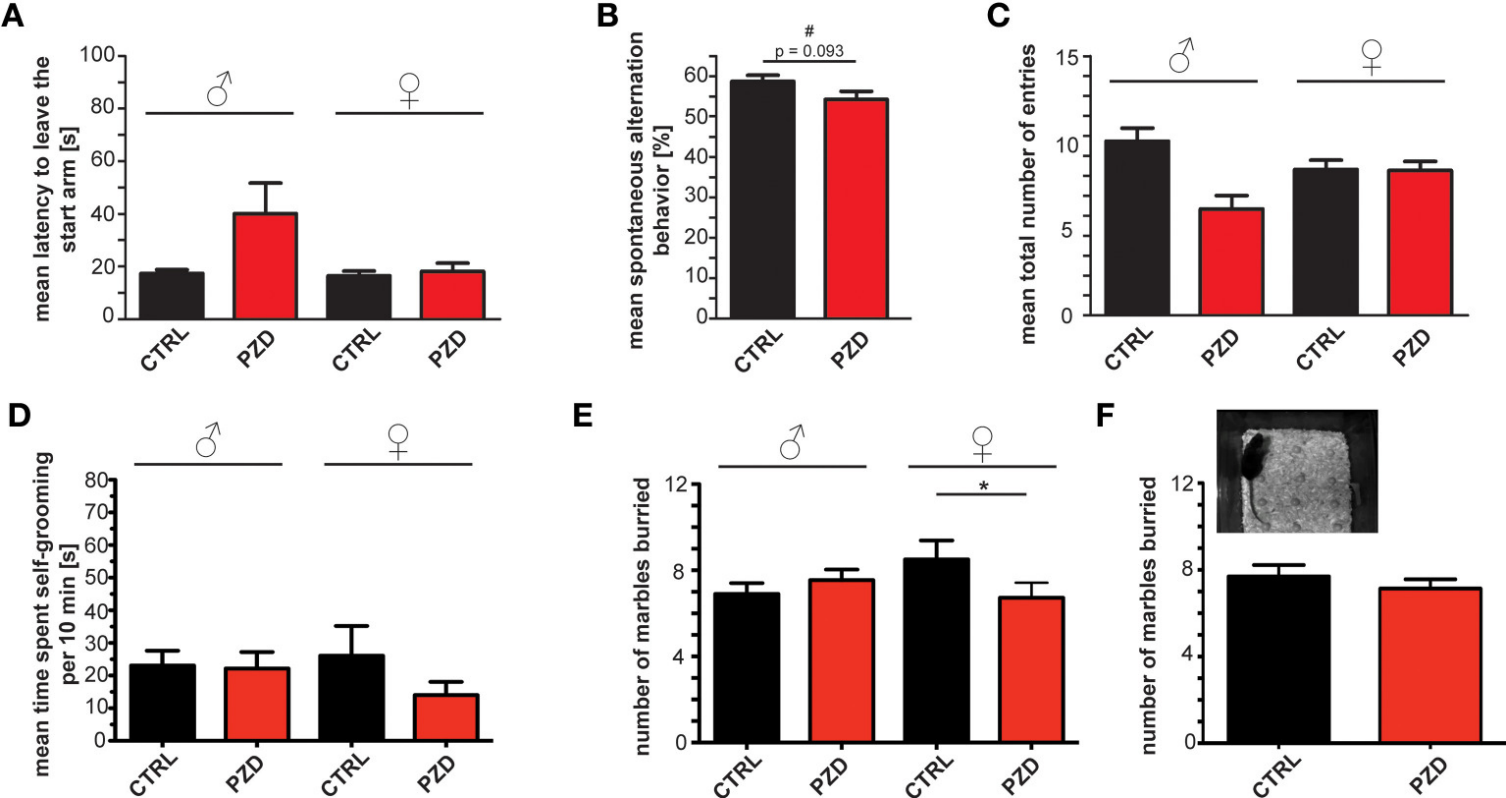
**PZD mice show only minor differences in spontaneous alternation and repetitive behavior. (A–C)** Spontaneous alternation behavior in the Y-maze labyrinth, a hippocampus dependent task of spatial working memory. **(A)** No significant differences in the latency to leave the starting arm were observed. **(B)** A trend (#) toward a decrease in the percentage of alternation during the 5 min test session in the Y maze labyrinth was detected in PZD mice. **(C)** The mean number of entries was not altered. **(A–C)** No gender specific effects were observed in the Y-maze test. **(D)** PZD mice show no significant differences in the time spent self-grooming independent of gender. *N* = 24 CTRL animals (12 males and 12 females) and *n* = 25 PZD mice (12 males and 13 females) were analyzed. **(E,F)** Repetitive marble burying behavior was measured in PZD mice after a 30 min test session. PZD females buried significantly less marbles in comparison to control mice. *N* = 20 CTRL animals (10 males and 10 females) and *n* = 22 PZD mice (11 males and 11 females) were analyzed. ^*^p ≤ 0.05.

Additionally, we assessed spontaneous alternation in the Y-maze in order to assess spontaneous working memory. In the Y-maze test, a trend toward significantly increased latency could be detected in the latency to leave starting arm (Figure 7A; two-way ANOVA, main effect of the treatment: *F*_(1, 47)_ = 3.028, *p* = 0.088) between PZD mice and control mice. We observed no significant difference in spontaneous alternation indicating normal working memory in PZD mice (Figure 7B) although a strong trend toward a decrease was present in PZD mice. No significant difference in the mean total number of entries was observed (Figure 7C).

Finally, repetitive behavior is another core feature of ASD. As reduction of alternations in the Y-maze test could also indicate a restricted behavioral pattern (Roullet and Crawley, 2011), we analyzed time spent self-grooming, and performed a marble burying test. We could not detect significant differences in the time PZD mice spent self-grooming (Figure 7D). In addition, repetitive marble burying behavior (Angoa-Pérez et al., 2013) was measured in PZD mice (Figures 7E,F, we found a significant interaction between treatment and gender two-way ANOVA: treatment × gender interaction: *F*_(1, 38)_ = 4.185, *p* = 0.048). *Post hoc* analysis revealed that PZD female mice buried less marbles in comparison to CTRL female mice (*p* < 0.022). In line with some ASD mouse models such as synapsin (Greco et al., 2013), Shank1 (Sungur et al., 2014) knockout mice, or mutant Shank3 mice (Kouser et al., 2013), our results show that PZD females buried significantly less marbles in comparison to control mice (Figure 7E).

## Discussion

Already in the first reports on human patients suffering from zinc deficiency described by Ananda Prasad in the early 1960s (Prasad et al., 1961, 1963a,b), among other symptoms, mental lethargy was noted. Since then, many studies on humans, but more so on animal models for zinc deficiency, have found specific impairments such as abnormal neurosensory changes, emotional instability, increased anxiety and aggression, irritability and deficits in social behavior that are most likely caused by an underlying dysfunction of zinc dependent processes in the brain (Hagmeyer et al., 2015). However, the diversity of these processes makes it challenging to investigate the specific molecular mechanism underlying a certain behavioral impairment. One such mechanism that has been proposed for possible ASD like behavior is the dysregulation of zinc dependent synaptic Shank scaffold proteins and of the zinc-metalloprotease-BDNF axis (Grabrucker, 2014; Koh et al., 2014).

Intriguingly, many infants diagnosed with autism display a marginal to severe zinc deficiency, suggesting a relationship of infantile zinc deficiency with autism (Yasuda et al., 2011; Yasuda and Tsutsui, 2013). Zinc deficiency is linked to copper overload, which is also reflected in the increase in copper levels in the blood of mice on zinc deficient diet in this study, and the alteration in the Cu/Zn ratio was reported to correlate with the severity of autism - associated symptoms (Faber et al., 2009; Russo et al., 2012; Li et al., 2014). Recent research proposes a model whereby maternal zinc deficiency leads to impairments in gut development in the offspring, thus extending the period of zinc deficiency due to insufficient absorption, but also leading to gastrointestinal problems such as a “leaky gut” and immune-reactions in the developing child (Vela et al., 2015).

Here, we use prenatal zinc deficient mice that only experienced zinc deficiency during pregnancy. On postnatal day 2 (PD2), pups were shifted to control mothers where zinc levels normalize within few days (Grabrucker et al., 2014). Still, when tested in adulthood, these mice displayed significant behavioral alterations summarized in Table 1. Given that we have reported before that prenatal zinc deficient mice show impairments in ultrasonic vocalization as well as reduced maternal behavior and increased aggression in a resident intruder test (Grabrucker et al., 2014), it is possible to summarize that indeed, many behavioral abnormalities characteristic for ASD mouse models are present in PZD animals (Table 2).

**Table 2:** **Summary of ASD like behavioral phenotypes of PZD mice**.

**ASD feature**	**Test paradigm**	**PZD mice behavior**
Impaired communication	Ultrasonic vocalizations male/female urine^*^	Impaired
	Vocalization in reciprocal social interaction	Impaired
Repetitive behavior	Self-grooming	Normal
	Marble burying	Reduced (females)
	Y-maze	Normal
Impaired social behavior	Nesting	Impaired
	Sociability and social novelty test	Abnormal
	Olfactory habituation test	Normal
	Reciprocal social interaction	Abnormal / increased aggression
	Maternal behavior^*^	Impaired
	Resident intruder^*^	Increased aggression
Anxiety	Open field	Increased
	Elevated plus maze	Increased
Activity	Open field	No hyperactivity
Cognitive impairment	Motor learning	Reduced

* *were tested in (Grabrucker et al., 2014)*.

Similarly to human patients, mouse models for ASD show variable phenotypes and not all behavioral abnormalities characteristic for ASD are present to the same extent. For example, while many ASD models show impaired social approach behavior, others have shown apparently normal social approach but exhibit other social abnormalities. This is the case for the Itgb3 (Carter et al., 2011) mouse model, which has normal sociability as measured by the three-chamber test but shows a deficit in social novelty preference.

In PZD mice, the core features of ASD—like behavior such as impaired vocalization and abnormal social behavior are present. The observed social impairments are highly influenced by increased anxiety and, probably as coping strategy in social situations, increased aggression in PZD mice. For example, the increased time male PZD mice spend in a chamber with a stranger mouse and their increased sniffing rates as response to social odors might be a reflection of their increased aggression rather than social interest. In line with this, female mice that display less territoriality show slightly decreased social interest in the 3-chamber setup and reduced maternal behavior (Grabrucker et al., 2014) after exposure to PZD. However, PZD mice show almost no abnormalities regarding repetitive behaviors, with the exception of female mice that show abnormalities in the marble burying test.

Nevertheless, PZD mice seem to represent a mouse models with non-genetically triggered ASD—like phenotype. Given that several candidate genes that are associated with ASD, such as metallothioneins (MTs), zinc transporter 5 (ZnT5; SLC30A5), Zrt- and Irt- like Protein 5 (ZIP5; SLC39A5), metal responsive transcription factor-1 (MTF1) (Serajee et al., 2004; Levy et al., 2011; O'Roak et al., 2011; Sanders et al., 2012) are involved in zinc homeostasis, one might speculate that prenatal zinc deficiency in humans might act as potent trigger in individuals with specific genetic susceptibility due to variants in these ASD candidate genes with otherwise low penetrance.

Our data further shows few gender specific effects of PZD such as stronger phenotypes regarding nest building, marble burying and social novelty in female PZD mice. One model could be the gender-dependent influence by maternal, placental, and fetal hormones on the developing brain. For example, developing testes are more active than ovaries, resulting in higher levels of circulating testosterone and estradiol in male than in female fetuses. Conversion of testosterone to estrogen is mediated by a zinc dependent aromatase that is inhibited by zinc (Om and Chung, 1996). Under zinc deficient conditions, disinhibition might promote the conversion and thus lower testosterone levels, which might affect female and male pups differently.

Taken together, here, using state of the art behavioral analyses to determine a possible ASD like phenotype of PZD mice, we could confirm the early reports on increased anxiety and aggression in PZD mice and rats. Moreover, we could narrow the initial description of altered emotionality (Hagmeyer et al., 2015) to specific ASD—like deficits in social behavior that are accompanied by reduced vocalizations in social situations.

More studies in future will be needed to reveal the underlying molecular mechanisms of the observed behavioral abnormalities. However, the association of low bioavailability of zinc with the occurrence of autism in humans might point to a significant influence of pre- and peri-natal zinc status on the development of the disorder.

## Author contributions

AG designed the study, performed data analysis and wrote the paper with SG and TB. SG performed the experiments and data analysis.

## Funding

TB was supported by the Innovative Medicines Initiative (IMI) Joint Undertaking under grant agreement n° 115300, resources of which are composed of financial contribution from the European Union's Seventh Framework Programme (FP7/2007-2013) and EFPIA companies' in kind contribution.

### Conflict of interest statement

The authors declare that the research was conducted in the absence of any commercial or financial relationships that could be construed as a potential conflict of interest. The reviewer LR and handling Editor declared their shared affiliation, and the handling Editor states that the process nevertheless met the standards of a fair and objective review.
