# Supplementary material for: Gender Dependent Evaluation of Autism like Behavior in Mice Exposed to Prenatal Zinc Deficiency
Source: Front Behav Neurosci. 2016 Mar 3;10:37. doi: 10.3389/fnbeh.2016.00037 (PMC4776245; doi:10.3389/fnbeh.2016.00037)
Supplement: Supplementary file 1 [file DataSheet1.PDF]

Supplementary Material

Gender dependent evaluation of autism like behavior in mice exposed to prenatal zinc deficiency

Stefanie Grabrucker, Tobias M. Boeckers, Andreas M. Grabrucker*

*** Correspondence:** Corresponding Author: andreas.grabrucker@uni-ulm.de

1 Supplementary Data

Supplementary Data 1 – Statistical analysis

Table 1

Parameter	Comparison	Results
Shirpa<br>**Body weight**<br>WT male: n = 10<br>WT female: n = 10<br>PZD male: n = 11<br>PZD female: n = 11	effect of prenatal treatment, gender	2-way ANOVA:<br>main effect of the treatment: $F_{(1,38)} = 9.189$ $p < 0.004$<br>main effect of the gender: $F_{(1,38)} = 60.874$ $p < 0.001$<br>treatment x gender interaction: $F_{(1,38)} = 0.556$ $p = 0.461$
Shirpa<br>**Body positions**	effect of prenatal treatment, gender	2-way ANOVA:<br>main effect of the treatment: $F_{(1,38)} = 1.225$ $p = 0.275$<br>main effect of the gender: $F_{(1,38)} = 1.137$ $p = 0.293$<br>treatment x gender interaction: $F_{(1,38)} = 0.721$ $p = 0.401$
Shirpa<br>**Defecation**	effect of prenatal treatment, gender	2-way ANOVA:<br>main effect of the treatment: $F_{(1,38)} = 0.027$ $p = 0.871$<br>main effect of the gender: $F_{(1,38)} = 1.169$ $p = 0.286$<br>treatment x gender interaction: $F_{(1,38)} = 1.159$ $p = 0.280$
Shirpa<br>**Transfer arousal**	effect of prenatal treatment, gender	2-way ANOVA:<br>main effect of the treatment: $F_{(1,38)} = 10.964$ $p < 0.002$<br>main effect of the gender: $F_{(1,38)} = 0.508$ $p = 0.480$<br>treatment x gender interaction: $F_{(1,38)} = 1.281$ $p = 0.65$
Shirpa<br>**Locomotor activity**	effect of prenatal treatment, gender	2-way ANOVA:<br>main effect of the treatment: $F_{(1,38)} = 0.435$ $p = 0.513$<br>main effect of the gender: $F_{(1,38)} = 0.334$ $p = 0.567$<br>treatment x gender interaction: $F_{(1,38)} = 0.010$ $p = 0.922$
Shirpa<br>**Gait**	effect of prenatal treatment, gender	2-way ANOVA:<br>main effect of the treatment: $F_{(1,38)} = 1.106$ $p = 0.300$<br>main effect of the gender: $F_{(1,38)} = 1.106$ $p = 0.300$<br>treatment x gender interaction: $F_{(1,38)} = 1.106$ $p = 0.300$
Shirpa<br>**Tail elevation**	effect of prenatal treatment, gender	2-way ANOVA:<br>main effect of the treatment: $F_{(1,38)} = 2.051$ $p = 0.160$<br>main effect of the gender: $F_{(1,38)} = 2.051$ $p = 0.160$<br>treatment x gender interaction: $F_{(1,38)} = 4.307$ $p = 0.045$
Shirpa<br>**Startle response**	effect of prenatal treatment, gender	2-way ANOVA:<br>main effect of the treatment: $F_{(1,38)} = 2.496$ $p = 0.122$<br>main effect of the gender: $F_{(1,38)} = 0.00$ $p = 1.000$<br>treatment x gender interaction: $F_{(1,38)} = 0.000$ $p = 1.000$
Shirpa<br>**Touch escape**	effect of prenatal treatment, gender	2-way ANOVA:<br>main effect of the treatment: $F_{(1,38)} = 0.901$ $p = 0.349$

		main effect of the gender: $F_{(1,38)}$ = 3. 284 p = 0.078<br>treatment x gender interaction: $F_{(1,38)}$ = 0.007 p = 0.932
Shirpa<br>**Grip strength forepaw**	effect of prenatal treatment, gender	2-way ANOVA:<br>main effect of the treatment: $F_{(1,38)}$ = 0.752 p = 0.391<br>main effect of the gender: $F_{(1,38)}$ = 3.307 p = 0.077<br>treatment x gender interaction: $F_{(1,38)}$ = 0.904 p = 0.348
Shirpa<br>**Grip strength forepaw and hindpaw**	effect of prenatal treatment, gender	2-way ANOVA:<br>main effect of the treatment: $F_{(1,38)}$ = 0.133 p = 0.718<br>main effect of the gender: $F_{(1,38)}$ = 1.018 p = 0.319<br>treatment x gender interaction: $F_{(1,38)}$ = 0.072 p = 0.790
Shirpa<br>**Positional passivity**	effect of prenatal treatment, gender	2-way ANOVA:<br>main effect of the treatment: $F_{(1,38)}$ = 0.133 p = 0.718<br>main effect of the gender: $F_{(1,38)}$ = 1.018 p = 0.319<br>treatment x gender interaction: $F_{(1,38)}$ = 0.072 p = 0.790
Shirpa<br>**Skin color**	effect of prenatal treatment, gender	2-way ANOVA:<br>main effect of the treatment: $F_{(1,38)}$ = 0.106 p = 0.300<br>main effect of the gender: $F_{(1,38)}$ = 1.106 p = 0.300<br>treatment x gender interaction: $F_{(1,38)}$ = 1.106 p = 0.300
Shirpa<br>**Evidence of biting**	effect of prenatal treatment, gender	2-way ANOVA:<br>main effect of the treatment: $F_{(1,38)}$ = 0.106 p = 0.300<br>main effect of the gender: $F_{(1,38)}$ = 1.106 p = 0.300<br>treatment x gender interaction: $F_{(1,38)}$ = 1.106 p = 0.300
Shirpa<br>**Vocalization**	effect of prenatal treatment, gender	2-way ANOVA:<br>main effect of the treatment: $F_{(1,38)}$ = 0.268 p = 0.608<br>main effect of the gender: $F_{(1,38)}$ = 0.112 p = 0.739<br>treatment x gender interaction: $F_{(1,38)}$ = 0.112 p = 0.739

Figure 1 (E)

Body weight	effect of prenatal treatment, gender	3-way mixed ANOVA:<br>treatment: $F_{(1,38)}$ = 10.511, $p < 0.003$<br>gender: $F_{(1,38)}$ = 96.826, $p < 0.001$<br>age: $F_{(6,228)}$ = 604.866, $p < 0.001$<br>treatment x gender interaction: $F_{(1,38)}$ = 0.190, p = 0.666<br>treatment x age interaction: $F_{(6,228)}$ = 2.456, $p < 0.044$<br>gender x age interaction: $F_{(6,228)}$ = 5.279, $p < 0.001$<br>treatment x sex x chamber interaction, $F_{(6,228)}$ = 1.605, p = 0.171

Figure 2

Open Field

Parameter	Comparison	Results
Open field<br>**(A) duration in center zone**<br>WT male: n = 10<br>WT female: n = 10<br>PZD male: n = 11<br>PZD female: n = 11	effect of prenatal treatment, gender	2-way ANOVA:<br>main effect of the treatment: $F_{(1,38)}$ = 7.049 $p < 0.012$<br>main effect of the gender: $F_{(1,38)}$ = 2.867 p = 0.099<br>treatment x gender interaction: $F_{(1,38)}$ = 0.285 p = 0.596
Open field<br>**(B) duration boarder zone**<br>WT male: n = 10<br>WT female: n = 10<br>PZD male: n = 11<br>PZD female: n = 11	effect of prenatal treatment, gender	2-way ANOVA:<br>main effect of the treatment: $F_{(1,38)}$ = 5.495 $p < 0.024$<br>main effect of the gender: $F_{(1,38)}$ = 1.629 p = 0.210<br>treatment x gender interaction: $F_{(1,38)}$ = 0.717 p = 0.402
Open field<br>**(C) # entries center zone**<br>WT male: n = 10<br>WT female: n = 10<br>PZD male: n = 11<br>PZD female: n = 11	effect of prenatal treatment, gender	2-way ANOVA:<br>main effect of the treatment: $F_{(1,38)}$ = 4.018 $p < 0.052$<br>main effect of the gender: $F_{(1,38)}$ = 0.183 p = 0.671<br>treatment x gender interaction: $F_{(1,38)}$ = 0.002 p = 0.966
Open field<br>**(D) distance travelled**	effect of prenatal treatment, gender	2-way ANOVA:<br>main effect of the treatment: $F_{(1,38)}$ = 0.811 p = 0.373

WT male: n = 10<br>WT female: n = 10<br>PZD male: n = 11<br>PZD female: n = 11		main effect of the gender: $F_{(1,38)} = 0.728$ $p = 0.399$<br>treatment x gender interaction: $F_{(1,38)} = 1.251$ $p = 0.270$
Open field<br>**(E) velocity**<br>WT male: n = 10<br>WT female: n = 10<br>PZD male: n = 11<br>PZD female: n = 11	effect of prenatal treatment, gender	2-way ANOVA:<br>main effect of the treatment: $F_{(1,38)} = 0.627$ $p = 0.433$<br>main effect of the gender: $F_{(1,38)} = 0.838$ $p = 0.366$<br>treatment x gender interaction: $F_{(1,38)} = 1.182$ $p = 0.284$
Open field<br>**(F) number of ambulations**<br>WT male: n = 10<br>WT female: n = 10<br>PZD male: n = 11<br>PZD female: n = 11	effect of prenatal treatment, gender	2-way ANOVA:<br>main effect of the treatment: $F_{(1,38)} = 2.231$ $p = 0.144$<br>main effect of the gender: $F_{(1,38)} = 8.685$ $p < 0.005$<br>treatment x gender interaction: $F_{(1,38)} = 0.095$ $p = 0.760$

**Parameter**	**Comparison**	**Results**
Elevated plus maze<br>**(G) time spent in open arms**<br>WT male: n = 22<br>WT female: n = 10<br>PZD male: n = 25<br>PZD female: n = 11	effect of prenatal treatment, gender	2-way ANOVA:<br>main effect of the treatment: $F_{(1,64)} = 5.335$ $p = 0.027$<br>main effect of the gender: $F_{(1,64)} = 1.064$ $p = 0.306$<br>treatment x gender interaction: $F_{(1,64)} = 0.080$ $p = 0.779$
Elevated plus maze<br>**(H) entries into closed arms**<br>WT male: n = 22<br>WT female: n = 10<br>PZD male: n = 25<br>PZD female: n = 11	effect of prenatal treatment, gender	2-way ANOVA:<br>main effect of the treatment: $F_{(1,64)} = 1.874$ $p = 0.176$<br>main effect of the gender: $F_{(1,64)} = 8.120$ $p < 0.006$<br>treatment x gender interaction: $F_{(1,64)} = 1.691$ $p = 0.198$
Elevated plus maze<br>**(I) entries into open arms**<br>WT male: n = 22<br>WT female: n = 10<br>PZD male: n = 25<br>PZD female: n = 11	effect of prenatal treatment, gender	2-way ANOVA:<br>main effect of the treatment: $F_{(1,64)} = 2.008$ $p = 0.161$<br>main effect of the gender: $F_{(1,64)} = 8.089$ $p < 0.006$<br>treatment x gender interaction: $F_{(1,64)} = 1.817$ $p = 0.182$
Elevated plus maze<br>**(J) total number of entries**<br>WT male: n = 22<br>WT female: n = 10<br>PZD male: n = 25<br>PZD female: n = 11	effect of prenatal treatment, gender	2-way ANOVA:<br>main effect of the treatment: $F_{(1,64)} = 0.076$ $p = 0.783$<br>main effect of the gender: $F_{(1,64)} = 0.018$ $p = 0.894$<br>treatment x gender interaction: $F_{(1,64)} = 4.454$ $p < 0.039$<br><br>planned comparison:<br>WT males vs PZD males: p = 0.214<br>WT females vs PZD females: p = 0.345<br>WT (males vs females): p = 0.114<br>PZD (males vs females): p = 0.177
Elevated plus maze<br>**(K) track length**<br>WT male: n = 22<br>WT female: n = 10<br>PZD male: n = 25<br>PZD female: n = 11	effect of prenatal treatment, gender	2-way ANOVA:<br>main effect of the treatment: $F_{(1,64)} = 4.989$ $p < 0.029$<br>main effect of the gender: $F_{(1,64)} = 0.417$ $p = 0.521$<br>treatment x gender interaction: $F_{(1,64)} = 1.013$ $p = 0.318$
Elevated plus maze<br>**(L) velocity**<br>WT male: n = 22<br>WT female: n = 10<br>PZD male: n = 25<br>PZD female: n = 11	effect of prenatal treatment, gender	2-way ANOVA:<br>main effect of the treatment: $F_{(1,64)} = 5.96$ $p < 0.017$<br>main effect of the gender: $F_{(1,64)} = 0.119$ $p = 0.731$<br>treatment x gender interaction: $F_{(1,64)} = 1.403$ $p = 0.241$
Elevated plus maze<br>**(M) ambulations**<br>WT male: n = 22<br>WT female: n = 10<br>PZD male: n = 25<br>PZD female: n = 11	effect of prenatal treatment, gender	2-way ANOVA:<br>main effect of the treatment: $F_{(1,64)} = 1.198$ $p = 0.278$<br>main effect of the gender: $F_{(1,64)} = 3.618$ $p = 0.062$<br>treatment x gender interaction: $F_{(1,64)} = 1.767$ $p = 0.189$

Figure 3

Parameter	Comparison	Results
Nest building<br>**(A) nest score**<br><br>WT male: n = 10<br>WT female: n = 10<br>PZD male: n = 11<br>PZD female: n = 11	WT vs PZD<br>female vs male<br><br>WT male, WT female, PZD male, PZD female	Mann-Whitney-U-Test: (U = 140 $p < 0.023$)<br>Mann-Whitney-U-Test: (U = 118 $p < 0.007$)<br><br>Kruskal Wallis ANOVA: chi-square: 11.323, df = 3 $p < 0.01$<br><br>Post hoc test:<br>WT males vs PZD males: p = 0.251<br>WT females vs PZD females: p = 0.049

Figure 4

Three Chamber Test (Fig. 4 A-I)

Parameter	Comparison	Chamber time	Sniffing time	Side preference
Habituation	WT male: 10	*within group repeated measures ANOVA* $F_{(1,9)} = 0.266, p = 0.618$		No
Habituation	PZD male: 11	*within group repeated measures ANOVA* $F_{(1,10)} = 0.077, p = 0.787$		No
Habituation	WT female: 10	*within group repeated measures ANOVA* $F_{(1,9)} = 0.001, p = 0.972$		No
Habituation	PZD female: 11	*within group repeated measures ANOVA* $F_{(1,10)} = 4.185, p = 0.068$		No

Parameter	Comparison	Results
Habituation<br>**(A) Time spent in chamber**<br>WT male: n = 10<br>WT female: n = 10<br>PZD male: n = 11<br>PZD female: n = 11	effect of prenatal treatment, gender, chamber side	3-way mixed ANOVA:<br>treatment: $F_{(1,38)} = 1.097, p = 0.156$<br>gender: $F_{(1,38)} = 1.338, p = 0.255$<br>chamber: $F_{(1,38)} = 0.127, p = 0.723$<br>treatment x gender: $F_{(1,38)} = 0.756, p = 0.390$<br>treatment x trial interaction: $F_{(4,172)} = 4.384, p = 0.004$<br>gender x chamber interaction: $F_{(1,38)} = 1.290, p = 0.263$<br>treatment x sex x trial interaction: $F_{(1,38)} = 0.273, p = 0.0605$
Habituation<br>**(B) Mean # of transitions**<br>WT male: n = 10<br>WT female: n = 10<br>PZD male: n = 11<br>PZD female: n = 11	effect of prenatal treatment, gender, chamber side	3-way mixed ANOVA:<br>treatment: $F_{(1,38)} = 16.004, p < 0.000$<br>gender: $F_{(1,38)} = 0.739, p = 0.395$<br>chamber: $F_{(1,38)} = 0.375, p = 0.248$<br>treatment x gender: $F_{(1,38)} = 0.739, p = 0.395$<br>treatment x trial interaction: $F_{(4,172)} = 0.069, p = 0.740$<br>no gender x chamber interaction, $F_{(1,38)} = 0.374, p = 0.545$<br>treatment x sex x chamber interaction, $F_{(1,38)} = 0.112, p = 0.740$

Parameter	Comparison	Chamber time	Sniffing time	Sociability
Sociability	WT male: 10	*within group repeated measures ANOVA* $F_{(1,9)} = 25.265, p < 0.001$	*within group repeated measures ANOVA* $F_{(1,9)} = 41.884, p < 0.001$	Present
Sociability	PZD male: 11	*within group repeated measures ANOVA* $F_{(1,10)} = 39.208, p < 0.001$	*within group repeated measures ANOVA* $F_{(1,10)} = 45.577, p < 0.001$	Present
Sociability	WT female: 10	*within group repeated measures ANOVA* $F_{(1,9)} = 6.518, p < 0.031$	*within group repeated measures ANOVA* $F_{(1,9)} = 27.170, p < 0.001$	Present
Sociability	PZD female: 11	*within group repeated measures ANOVA* $F_{(1,10)} = 10.935, p < 0.008$	*within group repeated measures ANOVA* $F_{(1,10)} = 32.438, p < 0.001$	Present

Parameter	Comparison	Results
Sociability<br>**(D) Time spent in chamber**<br>WT male: n = 10<br>WT female: n = 10<br>PZD male: n = 11<br>PZD female: n = 11	effect of prenatal treatment, gender, chamber side	3-way mixed ANOVA:<br>treatment: $F_{(1,38)} = 0.043, p < 0.837$<br>gender: $F_{(1,38)} = 0.975, p < 0.330$<br>chamber: $F_{(1,38)} = 74.725, p < 0.000$<br>treatment x gender: $F_{(1,38)} = 0.917, p = 0.344$<br>treatment x chamber interaction: $F_{(1,38)} = 0.309, p = 0.581$<br>gender x chamber interaction: $F_{(1,38)} = 9.082, p = 0.005$<br>treatment x sex x chamber interaction: $F_{(1,38)} = 0.076, p = 0.785$
Sociability	effect of prenatal	3-way mixed ANOVA:<br>treatment: $F_{(1,38)} = 3.794, p < 0.059$

**(E) Time spent sniffing**<br>WT male: n = 10<br>WT female: n = 10<br>PZD male: n = 11<br>PZD female: n = 11	treatment, gender, chamber side	gender: $F_{(1,38)}$ = 13.171, p < 0.001<br>chamber: $F_{(1,38)}$ = 153.072, p < 0.000<br>treatment x gender: $F_{(1,38)}$ = 4.418, p = 0.042<br>treatment x chamber interaction, $F_{(1,38)}$ = 2.522, p = 0.121<br>gender x chamber interaction, $F_{(1,38)}$ = 10.815, p = 0.002 treatment x sex x chamber interaction, $F_{(1,38)}$ = 2.358, p = 0.133
Sociability<br>**(F) Mean # of transitions**<br>WT male: n = 10<br>WT female: n = 10<br>PZD male: n = 11<br>PZD female: n = 11	effect of prenatal treatment, gender, chamber side	3-way mixed ANOVA:<br>treatment: $F_{(1,38)}$ = 2.705, p < 0.108<br>gender: $F_{(1,38)}$ = 1.060, p < 0.310<br>chamber: $F_{(1,38)}$ = 3.597, p < 0.066<br>no treatment x gender: $F_{(1,38)}$ = 1.344, p = 0.253<br>treatment x chamber interaction: $F_{(1,38)}$ = 0.844, p = 0.364<br>gender x chamber interaction: $F_{(1,38)}$ = 0.211, p = 0.649<br>treatment x sex x chamber interaction: $F_{(1,38)}$ = 2.239, p = 0.143

parameter	comparison	chamber time	sniffing time	Social novelty
Social Novelty	WT male: 10	*within group repeated measures ANOVA*<br>$F_{(1,9)}$ = 24.425, p < 0.001	*within group repeated measures ANOVA*<br>$F_{(1,9)}$ = 20.136, p < 0.002	Present
Social Novelty	PZD male: 11	*within group repeated measures ANOVA*<br>$F_{(1,10)}$ = 45.577, p < 0.001	*within group repeated measures ANOVA*<br>$F_{(1,10)}$ = 43.260, p < 0.001	Present
Social Novelty	WT female:10	*within group repeated measures ANOVA*<br>$F_{(1,9)}$ = 6.279, p < 0.033	*within group repeated measures ANOVA*<br>$F_{(1,9)}$ = 29.718, p < 0.001	Present
Social Novelty	PZD female: 11	*within group repeated measures ANOVA*<br>$F_{(1,10)}$ = 3.893, p < 0.077	*within group repeated measures ANOVA*<br>$F_{(1,10)}$ = 6.189, p < 0.035	Present

Parameter	Comparison	Results
Social Novelty<br>**(G) Time spent in chamber**<br>WT male: n = 10<br>WT female: n = 10<br>PZD male: n = 11<br>PZD female: n = 11	effect of prenatal treatment, gender, chamber side	3-way mixed ANOVA:<br>treatment: $F_{(1,38)}$ = 0.505, p < 0.482<br>gender: $F_{(1,38)}$ = 0.399, p < 0.531<br>chamber: $F_{(1,38)}$ = 41.658, p < 0.000<br>treatment x gender: $F_{(1,38)}$ = 2.235, p = 1.143<br>treatment x chamber interaction: $F_{(1,38)}$ = 0.309, p = 0.581<br>gender x chamber interaction: $F_{(1,38)}$ = 1.095, p = 0.302<br>treatment x sex x chamber interaction: $F_{(1,38)}$ = 2.108, p = 0.155
Social Novelty<br>**(H) Time spent sniffing**<br>WT male: n = 10<br>WT female: n = 10<br>PZD male: n = 11<br>PZD female: n = 11	effect of prenatal treatment, gender, chamber side	3-way mixed ANOVA:<br>treatment: $F_{(1,38)}$ = 9.948, p < 0.003<br>gender: $F_{(1,38)}$ = 3.643, p < 0.064<br>chamber: $F_{(1,38)}$ = 62.566, p < 0.000<br>treatment x gender: $F_{(1,38)}$ = 0.326, p = 0.571<br>treatment x chamber interaction: $F_{(1,38)}$ = 2.170, p = 0.149<br>gender x chamber interaction: $F_{(1,38)}$ = 1.268, p = 0.267<br>treatment x sex x chamber interaction: $F_{(1,38)}$ = 2.082, p = 0.157
Social Novelty<br>**(I) Mean # of transitions**<br>WT male: n = 10<br>WT female: n = 10<br>PZD male: n = 11<br>PZD female: n = 11	effect of prenatal treatment, gender, chamber side	3-way mixed ANOVA:<br>treatment: $F_{(1,38)}$ = 1.949, p < 0.171<br>gender: $F_{(1,38)}$ = 3.222, p < 0.081<br>chamber: $F_{(1,38)}$ = 10.540, p < 0.002<br>no treatment x gender: $F_{(1,38)}$ = 0.080, p = 0.778<br>treatment x chamber interaction: $F_{(1,38)}$ = 0.360, p = 0.552<br>gender x chamber interaction: $F_{(1,38)}$ = 0.383, p = 0.540<br>treatment x sex x chamber interaction: $F_{(1,38)}$ = 0.265, p = 0.609

Olfactory Habituation (Fig. 4 J,K)

	Hab. to water	Dishab. water 3 vs. almond 1	Hab. to almond	Dishab. almond 3 vs. banana 1	Hab. to banana	Dishab. banana 3 vs. social C1	Hab. to social C1	Dishab. social C3 vs. social B1	Hab. to social B1
**WT** male **n = 10**	*within group repeated measures ANOVA* $F_{(2,18)}$ = 7.565, $p <$ 0.007	*within group repeated measures ANOVA* $F_{(1,9)}$ = 10.855, $p <$ 0.011	*within group repeated measures ANOVA* $F_{(2,18)}$ = 14.635, $p <$ 0.004	*within group repeated measures ANOVA* $F_{(1,9)}$ = 46.161, $p <$ 0.001	*within group repeated measures ANOVA* $F_{(2,18)}$ = 36.259, $p <$ 0.001	*within group repeated measures ANOVA* $F_{(1,9)}$ = 49.069, $p <$ 0.001	*within group repeated measures ANOVA* $F_{(2,18)}$ = 10.563, $p <$ 0.003	*within group repeated measures ANOVA* $F_{(1,9)}$ = 7.241, $p <$ 0.027	*within group repeated measures ANOVA* $F_{(2,18)}$ = 8.400, $p <$ 0.003
**PZD** male **n = 11**	*within group repeated measures ANOVA* $F_{(2,20)}$ = 5.401, $p <$ 0.030	*within group repeated measures ANOVA* $F_{(1,10)}$ = 11.955, $p <$ 0.006	*within group repeated measures ANOVA* $F_{(2,20)}$ = 17.177, $p <$ 0.001	*within group repeated measures ANOVA* $F_{(1,10)}$ = 23.315, $p <$ 0.001	*within group repeated measures ANOVA* $F_{(2,20)}$ = 29.573, $p <$ 0.001	*within group repeated measures ANOVA* $F_{(1,10)}$ = 50.032, $p <$ 0.001	*within group repeated measures ANOVA* $F_{(2,20)}$ = 10.390, $p <$ 0.002	*within group repeated measures ANOVA* $F_{(1,10)}$ = 17.335, $p <$ 0.002	*within group repeated measures ANOVA* $F_{(2,20)}$ = 25.279, $p <$ 0.001
**WT** female **n = 10**	*within group repeated measures ANOVA* $F_{(2,18)}$ = 2.878, $p =$ 0.107	*within group repeated measures ANOVA* $F_{(1,9)}$ = 4.527, $p <$ 0.062	*within group repeated measures ANOVA* $F_{(2,18)}$ = 5.443, $p <$ 0.034	*within group repeated measures ANOVA* $F_{(1,9)}$ = 13.943 $p <$ 0.005	*within group repeated measures ANOVA* $F_{(2,18)}$ = 21.608, $p <$ 0.001	*within group repeated measures ANOVA* $F_{(1,9)}$ = 99.452 $p <$ 0.001	*within group repeated measures ANOVA* $F_{(2,18)}$ = 18.377, $p <$ 0.001	*within group repeated measures ANOVA* $F_{(1,9)}$ = 6.554 $p <$ 0.031	*within group repeated measures ANOVA* $F_{(2,18)}$ = 7.110, $p <$ 0.012
**PZD** female **n = 11**	*within group repeated measures ANOVA* $F_{(2,20)}$ = 0.910, $p =$ 0.380	*within group repeated measures ANOVA* $F_{(1,10)}$ = 6.540, $p <$ 0.028	*within group repeated measures ANOVA* $F_{(2,20)}$ = 43.038, $p <$ 0.000	*within group repeated measures ANOVA* $F_{(1,10)}$ = 3.578, $p <$ 0.081	*within group repeated measures ANOVA* $F_{(2,20)}$ = 3.995, $p <$ 0.035	*within group repeated measures ANOVA* $F_{(1,10)}$ = 60.579, $p <$ 0.001	*within group repeated measures ANOVA* $F_{(2,20)}$ = 23.092, $p <$ 0.001	*within group repeated measures ANOVA* $F_{(1,10)}$ = 8.984, $p <$ 0.013	*within group repeated measures ANOVA* $F_{(2,20)}$ = 2.693, $p <$ 0.092

WT male: 10<br>PZD male: 11	Sniffing across **social** odors (social C, B) (peak height)	effect on prenatal treatment	Repeated measures ANOVA: treatment, $F_{(1,19)}$ = 4.804, $p <$ 0.042
WT male: 10<br>PZD male: 11	Sniffing across **non social** odors (almond, banana) (peak height)	effect on prenatal treatment	Repeated measures ANOVA: treatment, $F_{(1,19)}$ = 0.817, $p =$ 0.378
WT female:10<br>PZD female: 11	Sniffing across **social** odors (social C, B) (peak height),	effect on prenatal treatment	Repeated measures ANOVA: treatment, $F_{(1,19)}$ = 0.031, $p =$ 0.861
WT female:10<br>PZD female: 11	Sniffing across **non social** odors (social C, B) (peak height)	effect on prenatal treatment	Repeated measures ANOVA: treatment, $F_{(1,19)}$ = 0.230, $p =$ 0.673

Resident Intruder (Fig. 4 L-O)

**Parameter**	**Comparison**	**Results**
Resident Intruder<br>**(M) Time spent in contact**<br>WT male: n = 10<br>WT female: n = 10<br>PZD male: n = 11<br>PZD female: n = 11	effect of prenatal treatment, gender	2-way ANOVA:<br>main effect of the treatment: $F_{(1,38)} = 0.003$ $p = 0.960$<br>main effect of the gender: $F_{(1,38)} = 2.901$ $p = 0.097$<br>treatment x gender interaction: $F_{(1,38)} = 0.194$ $p = 0.662$
Resident Intruder	effect of prenatal	2-way ANOVA:

**(N) Time spent in contact (oral-oral)**<br>WT male: n = 10<br>WT female: n = 10<br>PZD male: n = 11<br>PZD female: n = 11	treatment, gender	main effect of the treatment: $F_{(1,38)} = 15.103$ $p = 0.000$<br>main effect of the gender: $F_{(1,38)} = 8.352$ $p = 0.006$<br>treatment x gender interaction: $F_{(1,38)} = 5.335$ $p = 0.027$<br><br>planned comparison:<br>WT male vs PZD male: p = 0.687<br>WT female vs PZD female: p < 0.004<br>WT (males vs females): p = 0.568<br>PZD (males vs females): p < 0.009
Resident Intruder<br>**(O) Resident behind intruder**<br>WT male: n = 10<br>WT female: n = 10<br>PZD male: n = 11<br>PZD female: n = 11	effect of prenatal treatment, gender	2-way ANOVA:<br>main effect of the treatment: $F_{(1,38)} = 3.582$ $p < 0.038$<br>main effect of the gender: $F_{(1,38)} = 0.004$ $p = 0.950$<br>treatment x gender interaction: $F_{(1,38)} = 1.135$ $p = 0.294$<br><br>WT Male vs PZD Male p = 0.598<br>WT Male vs PZD Female p = 0.107

Figure 5

Vocalization<br>**(A) Latency to call**<br>WT male: n = 10<br>WT female: n = 10<br>PZD male: n = 11<br>PZD female: n = 11	effect of prenatal treatment, gender	2-way ANOVA:<br>main effect of the treatment: $F_{(1,38)} = 5.215$ $p = 0.028$<br>main effect of the gender: $F_{(1,38)} = 1.399$ $p = 0.244$<br>treatment x gender interaction: $F_{(1,38)} = 0.280$ $p = 0.600$
Vocalization<br>**(B) Total number of calls**<br>WT male: n = 10<br>WT female: n = 10<br>PZD male: n = 11<br>PZD female: n = 11	effect of prenatal treatment, gender	2-way ANOVA:<br>main effect of the treatment: $F_{(1,38)} = 0.011$ $p = 0.918$<br>main effect of the gender: $F_{(1,38)} = 0,343$ $p = 0.562$<br>treatment x gender interaction: $F_{(1,38)} = 0.427$ $p = 0.871$

Figure 6

Rotarod<br>**(A,B) Latency to fall**<br>WT male: n = 13<br>WT female: n = 9<br>PZD male: n = 14<br>PZD female: n = 10	effect of prenatal treatment, gender	Three-way mixed ANOVA:<br>treatment, $F_{(1,41)} = 5.116$, $p < 0.029$<br>gender, $F_{(1,41)} = 21.609$, $p < 0.001$<br>trial, $F_{(4,168)} = 13.077$, $p < 0.001$<br>treatment x gender, $F_{(1,43)} = 1.536$, $p = 0.222$<br>treatment x trial interaction, $F_{(4,168)} = 3.750$, $p < 0.010$<br>gender x trial interaction, $F_{(4,168)} = 1.868$, $p = 0.132$<br>treatment x sex x trial interaction, $F_{(4,164)} = 0.225$, $p = 0.924$

Figure 7

Parameter	Comparison	Results
Y Maze<br>**(A) Latency to leave start arm**<br>WT male: n = 12<br>WT female: n = 12<br>PZD male: n = 12<br>PZD female: n = 13	effect of prenatal treatment, gender	2-way ANOVA:<br>main effect of the treatment: $F_{(1,47)} = 3.028$ $p = 0.088$<br>main effect of the gender: $F_{(1,47)} = 2.619$ $p = 0.112$<br>treatment x gender interaction: $F_{(1,47)} = 2.742$ $p = 0.104$
Y Maze<br>**(B) Spontaneous alternation**<br>WT male: n = 12<br>WT female: n = 12<br>PZD male: n = 12<br>PZD female: n = 13	effect of prenatal treatment, gender	2-way ANOVA:<br>main effect of the treatment: $F_{(1,47)} = 2.936$ $p = 0.093$<br>main effect of the gender: $F_{(1,47)} = 2.476$ $p = 0.112$<br>treatment x gender interaction: $F_{(1,47)} = 0.165$ $p = 0.686$

Y Maze<br>**(C) Number of arm entries**<br>WT male: n = 12<br>WT female: n = 12<br>PZD male: n = 12<br>PZD female: n = 13	effect of prenatal treatment, gender	2-way ANOVA:<br>main effect of the treatment: $F_{(1,47)} = 3.165$ $p = 0.082$<br>main effect of the gender: $F_{(1,47)} = 5.515$ $p < 0.023$<br>treatment x gender interaction: $F_{(1,47)} = 2.776$ $p = 0.102$

**Parameter**	**Comparison**	**Results**
Grooming<br>**(D) Time spent grooming**<br>WT male: n = 10<br>WT female: n = 10<br>PZD male: n = 11<br>PZD female: n = 11	effect of prenatal treatment, gender	2-way ANOVA:<br>main effect of the treatment: $F_{(1,38)} = 1.882$ $p = 0.178$<br>main effect of the gender: $F_{(1,38\ )} = 0.326$ $p = 0.571$<br>treatment x gender interaction: $F_{(1,38)} = 1.090$ $p = 0.303$
Marble Burying<br>**(E,F) # of marbles buried**<br>WT male: n = 10<br>WT female: n = 10<br>PZD male: n = 11<br>PZD female: n = 11	effect of prenatal treatment, gender	2-way ANOVA:<br>main effect of the treatment: $F_{(1,38)} = 0.841$ $p = 0.365$<br>main effect of the gender: $F_{(1,38\ )} = 0.434$ $p = 0.514$<br>treatment x gender interaction: $F_{(1,38)} = 4.185$ $p = 0.048$<br><br>WT males vs PZD males: p = 0.529<br>WT females vs PZD females: p = 0.022<br>WT (males vs females): p = 0.111<br>PZD (males vs females): p = 0.177
